# Genome-Wide SNP Analysis of Hybrid Clariid Fish Reflects the Existence of Polygenic Sex-Determination in the Lineage

**DOI:** 10.3389/fgene.2022.789573

**Published:** 2022-02-03

**Authors:** Dung Ho My Nguyen, Jatupong Ponjarat, Nararat Laopichienpong, Thitipong Panthum, Worapong Singchat, Syed Farhan Ahmad, Ekaphan Kraichak, Narongrit Muangmai, Prateep Duengkae, Surin Peyachoknagul, Uthairat Na-Nakorn, Kornsorn Srikulnath

**Affiliations:** ^1^ Animal Genomics and Bioresource Research Center (AGB Research Center), Faculty of Science, Kasetsart University, Bangkok, Thailand; ^2^ Laboratory of Animal Cytogenetics and Comparative Genomics (ACCG), Department of Genetics, Faculty of Science, Kasetsart University, Bangkok, Thailand; ^3^ Special Research Unit for Wildlife Genomics (SRUWG), Department of Forest Biology, Faculty of Forestry, Kasetsart University, Bangkok, Thailand; ^4^ Department of Botany, Kasetsart University, Bangkok, Thailand; ^5^ Department of Fishery Biology, Faculty of Fisheries, Kasetsart University, Bangkok, Thailand; ^6^ Department of Aquaculture, Faculty of Fisheries, Kasetsart University, Bangkok, Thailand; ^7^ Academy of Science, The Royal Society of Thailand, Bangkok, Thailand; ^8^ Center of Excellence on Agricultural Biotechnology (AG-BIO/PERDO-CHE), Bangkok, Thailand; ^9^ Amphibian Research Center, Hiroshima University, Higashihiroshima, Japan

**Keywords:** catfish, hybrid, SNP, polygenic sex-determination system, PSD

## Abstract

The African catfish (*Clarias gariepinus*) may exhibit the co-existence of XX/XY and ZZ/ZW sex-determination systems (SDSs). However, the SDS of African catfish might be influenced by a polygenic sex-determination (PSD) system, comprising multiple independently segregating sex “switch” loci to determine sex within a species. Here, we aimed to detect the existence of PSD using hybrid. The hybrid produced by crossing male African catfish with female bighead catfish (*C. macrocephalus*, XX/XY) is a good animal model to study SDSs. Determining the SDS of hybrid catfish can help in understanding the interactions between these two complex SDS systems. Using the genotyping-by-sequencing “DART-seq” approach, we detected seven moderately male-linked loci and seventeen female-linked loci across all the examined hybrid specimens. Most of these loci were not sex-linked in the parental species, suggesting that the hybrid exhibits a combination of different alleles. Annotation of the identified sex-linked loci revealed the presence of one female-linked locus homologous with the *B4GALNT1* gene, which is involved in the spermatogenesis pathway and hatchability. However, this locus was not sex-linked in the parental species, and the African catfish might also exhibit PSD.

## Introduction

Sex determination is a basic process in the evolution of sexual reproduction and diverse sex-determination systems (SDSs). Genetic sex determination systems, multifactorial polygenic sex-determining mechanisms, and environmental sex determination systems are found across taxa (Ezaz et al., 2016). Teleosts show a large diversity of SDSs, whereas turnovers or transitions often occur in many vertebrates (Pennell et al., 2018). ZZ/ZW and XX/XY are the common SDSs in the female and male heterogametic systems, and they have evolved repeatedly and independently in the lineage. This results in the presence of rapidly evolving SDSs in closely related species (Gammerdinger and Kocher, 2018; Nguyen et al., 2021a; Nguyen et al., 2021b). Thus, a study of sister taxa retaining divergent sex chromosome systems would be informative when examining SDS evolution. Among the 115 species of clariid fish (Clariidae), some have either male heterogametic or female heterogametic sex determination systems (Pandey and Lakra, 1997; Nguyen et al., 2021a). Both heterogametic systems are present together, including multiple loci and sex chromosomes in the lineage. Varying SDSs have been observed in African catfish (*Clarias gariepinus*, Burchell, 1822) based on their geographical location: a ZZ/ZW system in Africa, a XX/XY system is indicated in some populations from Israel, Hungary, and China, or both systems in the same population in Thailand (Teugels et al., 1992; Eding et al., 1997; Eyo and Effiong, 2005; Nguyen et al., 2021a). The SDS of African catfish might be influenced by a multifactorial, polygenic sex-determination (PSD) system that occurs in varied species. The occurrence of natural, multiple and independent “switch” loci or alleles specify the segregation of sex within a species. PSD can also occur by modifying current sex chromosomes to generate a third functional sex chromosome at the same locus, or by modifying autosomal loci in other regions of the genome to generate a novel process for gonad development regulation. A well-known example is observed in platyfish (*Xiphophorus maculatus*, Günther, 1866), with some populations showing segregation of male Y chromosome alleles and female W chromosome alleles at the same chromosome pair (Volff and Schartl, 2001). However, the genetic architecture contributing to variations in SDSs is not completely understood, especially in the few known examples of PSD in teleosts (Roberts et al., 2016). Clarification of the genetic structure of SDSs is essential to understand the evolutionary mechanisms and their influence on the processes of speciation (Qvarnström and Bailey, 2009), particularly in species without heteromorphic sex chromosomes.

Interspecific hybridization has successfully improved the growth rate, body size, disease resistance, and tolerance to stressors in aquaculture (Suprapto et al., 2017). For instance, male African catfish (*C. gariepinus*, 2n = 56) have been crossed with female bighead catfish (*Clarias macrocephalus*, Günther, 1864, 2n = 54 with XX/XY) to produce fast growth and improved disease resistance traits in the resulting hybrids (2n = 55) (Na-Nakorn, 1995; Chaivichoo et al., 2020; Nguyen et al., 2021a; Nguyen et al., 2021b). However, reproductive failure can occur as a result of chromosomal incompatibility with parental genomes. This can culminate in spermatogenic breakdown in F_1_ male hybrids, leading to subsequent elimination by apoptosis and limiting mass production (Ponjarat et al., 2019). By contrast, F_1_ female hybrids can produce copious backcross offspring with low embryo mortality (Na-Nakorn et al., 2004; Abol-Munafi et al., 2006) due to differences in the checkpoint systems in meiotic cells. This may be more stringent during the development of mature spermatozoa than production or development of an ovum or as a result of complex interactions between two different SDSs (Ponjarat et al., 2019). Thus, the male *C. gariepinus* × female *C. macrocephalus* hybrid is a good animal model to study the evolutionary process of SDSs in teleosts. In this study, we proposed the following two hypotheses: 1) the hybrid might possess the dominant SDS from male African catfish when Y or W sex chromosomes is present in different individuals, or 2) the hybrid might exhibit new candidate loci or a combination of different alleles due to the influence of PSD found in male African catfish. To determine how sex determination affects the viability of hybrid catfish, genome-wide single-nucleotide polymorphism (SNP) analyses were performed using Diversity Arrays Technology (DArTseq™) of captive-bred individuals scored with phenotypic sex, as described by Nguyen et al. (2021a) and Nguyen et al. (2021b). Results will provide insights to improve the understanding of SDSs in clariid fish.

## Materials and Methods

Adult individuals were collected as 15 male and 15 female hybrids, chosen randomly from a breeding stock (Kasetsart University, Bangkok, Thailand) to reduce the possibility of a high occurrence of siblings in the genetic pool. The hybrids were produced from pure breeding stock maintained for 10 years. Six generations of the breeding stock were bred in captivity. The sex of each individual was identified based on external morphology and internal examination of gonadal morphology (Esmaeili et al., 2017; Ponjarat et al., 2019). Animal care and all experimental procedures were approved by the Animal Experiment Committee, Kasetsart University, Thailand (Approval no. ACKU61-SCI-026) and concurred with the Regulations on Animal Experiments at Kasetsart University. The dorsal fins of each individual were removed for DNA extraction and total genomic DNA. DArT sequencing and genotyping, marker selection, DArT sequencing analysis, estimation of expected sex-linked markers, comparison of potential sex-linked loci, and homology searching processes were performed.

### Total Genomic DNA

Total genomic DNA was extracted following the salting-out protocol (Supikamolseni et al., 2015). The quality of each extracted DNA specimen was assessed using gel electrophoresis for the presence of high-molecular-weight DNA. All samples were stored at −20°C until required for DArTseq™ library construction (Laopichienpong et al., 2021).

### DArT Sequencing and Genotyping

Genotyping of multiple SNP loci was accomplished using Diversity Arrays Technology Pty Ltd. (DArTseq™) in Canberra, ACT, Australia following the methodology of Jaccoud et al. (2001) and Koomgun et al. (2020). The candidate sex-specific loci among male and female individuals were then determined using *in silico* DArT (variability in SNP loci generates presence/absence polymorphism in restriction sites, so-called PA markers). Approximately 100 ng of total DNA from each sample was used to develop the DArTseq™ arrays. The DNA samples were subjected to digestion and ligation reactions, as described by Kilian et al. (2012) and digested using *Pst*I and *Sph*I. The ligation reactions were performed using two adaptors as a *Pst*I compatible adaptor including an Illumina flow-cell attachment sequence, a sequencing primer and a unique barcode sequence, and a *Sph*I compatible adaptor consisting of an Illumina flow-cell attachment region. The ligated fragments were then processed by 30 cycles of PCR (94°C for 20 s, 58°C for 30 s, and 72°C for 45 s), with a final extension step at 72°C for 7 min. Equimolar amounts of amplification products from each individual were pooled and applied to Illumina’s proprietary cBot (http://www.illumina.com/products/cbot.html) bridge PCR, followed by sequencing on the Illumina HiSeq 2000 platform. The single read sequencing was run for 77 cycles. Sequences were processed using proprietary DArTseq analytical pipelines (Ren et al., 2015). Poor-quality sequences were filtered by processing the HiSeq 2000 output (FASTQ file). Two quality thresholds were applied to the barcode region for stringent selection as minimum Phred pass score of 30, and a 75% minimum pass length to allow parsing of sequences into specific sample libraries. Relaxed thresholds were applied to the remainder of the sequence as minimum Phred pass score 10 and minimum pass length 50%. Approximately 2,000,000 sequences per barcode/individual were recognized and used in marker calling. Finally, identical sequences were combined into “fastqcoll files” and used in the secondary pipeline (DArTsoft14) for proprietary SNP and PA loci calling, with the “reference-free” algorithm implemented in DArTsoft14. Sequence clusters were then parsed into SNP and *in silico* DArTseq™ markers utilizing a range of metadata parameters derived from the quantity and distribution of each sequence across all analyzed samples. Multiple libraries of the same individual were included in the DArTseq™ genotyping process, enabling reproducibility scores to be calculated for each candidate marker. The outputs generated by DArTsoft14 included reproducibility values at >90%, with read depth >3.5 for SNPs and >5 for PA markers, with call rate >80% (proportion of samples for which the marker was scored) (Koomgun et al., 2020; Laopichienpong et al., 2021; Nguyen et al., 2021a; Nguyen et al., 2021b).

### Marker Selection and DArT Sequencing Analysis

Sex-specific loci were derived from the analysis of SNP codominant markers and PA dominant markers. The SNP data were scored for homozygotes to reference allele as “0” (the most common allele), the alternate SNP allele homozygote as “1”, the heterozygote as “2”, or a score of “–” as the double null/null allele (absence of a fragment remaining the SNP in the genomic representation). The PA data were scored for presence as “1”, absence as “0” or a result of “–” for putative heterozygosity. For sex-linked markers in an XX/XY system, reference alleles are often found on the X-chromosome. Here, “SNP alleles” were those that showed polymorphism correlated to the reference allele. SNP alleles from an XX/XY system should be linked with the Y- chromosome and located on or near to the male-determination region if the allele is tightly Y-specific. If the two sex chromosomes recombine in an XX/XY sex chromosome system, SNP alleles should occasionally appear on the X chromosome. If this scenario occurs, then some males might be homozygous for SNP alleles at particular loci. As a result, females could be heterozygous and exhibit a copy of the SNP allele. However, this probability is low. Sex-linked or sex-specific loci were obtained from the analysis of SNP co-dominant markers and PA-dominant markers. For an XX/XY sex chromosome system, the SNP and PA loci sequenced for at least 70%, 80%, 90% and 100% of males were involved in a separate data set. Loci, where all males passed the 100% filtering criterion, were designated as perfectly male-linked, whereas those that passed the 70%–90% criterion were considered moderately male-linked loci (Koomgun et al., 2020; Laopichienpong et al., 2021; Nguyen et al., 2021a; Nguyen et al., 2021b). A similar approach was used to target loci with ZZ/ZW system. The Hamming distance was calculated as the number of pairwise differences between male and female individuals across SNP and PA loci using the “rdist” function in R software (R version 3.5.1). Heatmaps were represented using the function ‘pheatmap’ in ggplot2 R package (R Core Team, 2021). To evaluate the genetic association between each locus and phenotypic sex, we performed the Cochran-Armitage test (CATT) (Lambert et al., 2016; Sopniewski et al., 2019; Koomgun et al., 2020; Laopichienpong et al., 2021; Panthum et al., 2021). Similarly, the chi-square test was used to assess whether the proportion of different genotypes followed the null expectation. Polymorphism information content (*PIC*) values, as an index for evaluating SNP and PA loci, were calculated for each locus. The *PIC* values ranged from 0 (fixation of one allele) to 0.5 (frequencies of both alleles are equal) (Sven and Klaus, 2019; Koomgun et al., 2020; Laopichienpong et al., 2021; Nguyen et al., 2021a; Nguyen et al., 2021b; R Core Team, 2021).

### Estimation of Expected Sex-Linked Markers

The probability of random candidate sex-linked loci showing associations with sex under a small sample size was assessed using the formula *P*_i_ = 0.5^*n*^, where *P* is the probability that a given locus, *i* is sex-linked by chance, 0.5 is the probability that either a female is homozygous or a male is heterozygous at a given locus, and *n* is the number of individuals sequenced (Koomgun et al., 2020; Laopichienpong et al., 2021; Nguyen et al., 2021a; Nguyen et al., 2021b).

### Comparison of Potential Sex-Linked Loci

We designated all candidate loci, in which males (in the case of XY) or females (in the case of ZW) were not 100%, to moderately sex-linked loci: 90/10; 80/20; 70/30. Significant differences among the three groups of sex-linked loci were evaluated by the chi-square test using the R package “stats” for PA loci and the Kruskal–Wallis and Nemenyi test using the R package “PMCMR” for SNP loci (R Core Team, 2021), based on the mean heterozygosity and standard deviation for each. All candidate loci were plotted against each individual using the “glPlot” function in the dartR R package (Gruber and Georges, 2021; R Core Team, 2021).

### Homology Searches

For all sex-linked loci that met our criteria and had a statistically significant association with phenotypic sex, the hybrid sex-linked loci were searched to find the genome-wide SNP of their parents (*Clarias gariepinus* × *Clarias macrocephalus*) and detect the inherited loci. In this study, genome-wide SNP of *Clarias gariepinus* × *Clarias macrocephalus* was used from our previous study as described by Nguyen et al. (2021a); Nguyen et al. (2021b). NCBI BLAST was performed to discover homologies of sex-linked SNP/PA loci against a selection of available teleost fish genomes including Japanese rice fish (*Oryzias latipes*, Temminck and Schlegel, 1846), zebrafish (*Danio rerio*, Hamilton, 1822), Japanese pufferfish (*Takifugu rubripes*, Temminck and Schlegel, 1850), channel catfish (*Ictalurus punctatus*, Rafinesque, 1818), and chicken (*Gallus*, Linnaeus, 1758) (Supplementary Table S1). These species were selected as representative of reference genomes due to the availability of high-quality gene annotations and almost complete up-to-date assemblies (Takeda, 2008; Warren et al., 2017). A BLAST homology search was performed in two rounds. First, we aligned sex-linked loci against the reference teleost genome, and then mapped the homologous genes to further clarify their location on sex chromosomes of high-quality annotated genomes that were representative of vertebrate species. Sex-linked loci were used to search against both the NCBI “nr” (http://blast.ncbi.nlm.nih.gov/Blast.cgi) and the RepBase version 19.11 database (Genetic Information Research Institute; https://www.girinst.org/) (Bao et al., 2015). RepBase is a database of transposable elements including DNA transposons, LTR and non-LTR *retrotransposons* and endogenous retroviruses (ERVs). We chose criteria of E-values lower than .005 and query coverage with similarity of more than 60%.

## Results

**FIGURE 1:**
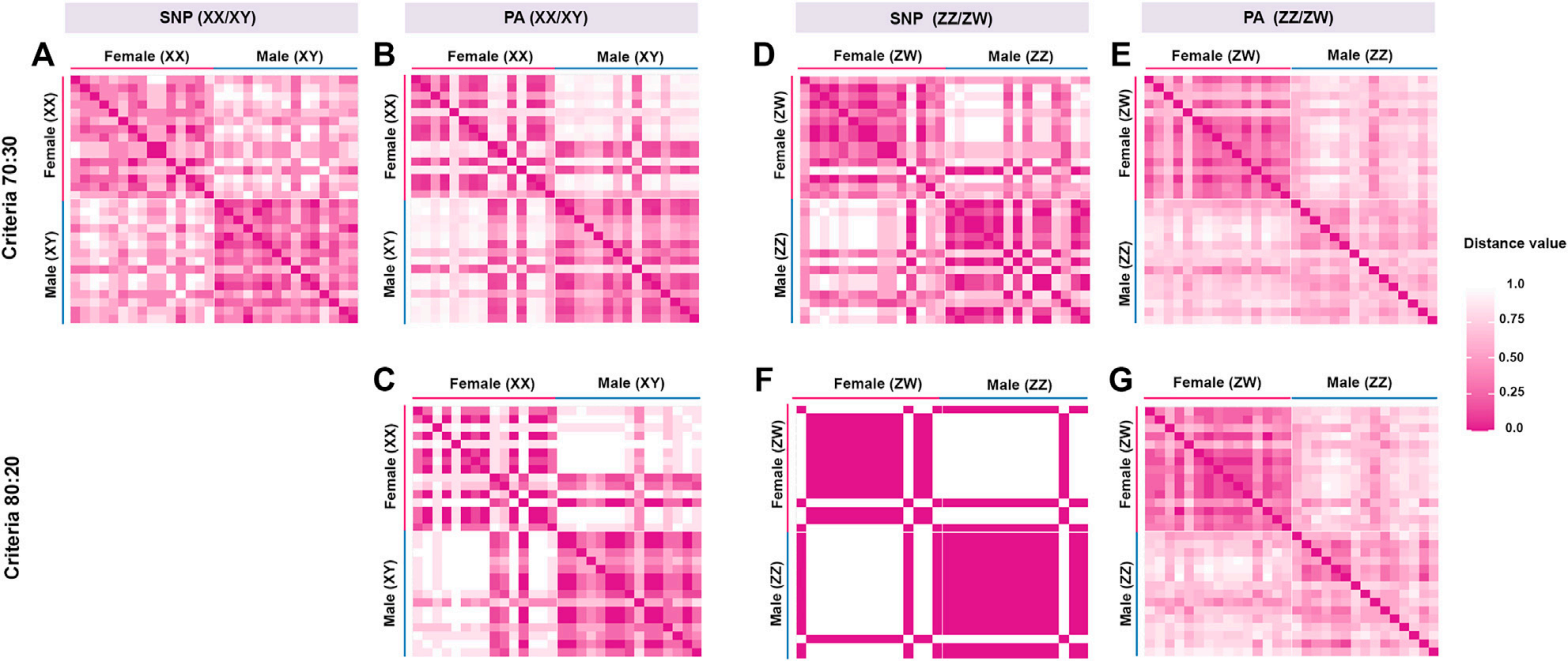
Hamming distances between male and female individuals of hybrid catfish (*Clarias gariepinus*, Burchell, 1822 x *Clarias macrocephalus*, Günther, 1864). The loci were filtered under different criteria. **(A)** Single-nucleotide polymorphic (SNP) loci filtered under the criterion 70:30 (male:female), **(B)** Restriction fragment presence/absence (PA) loci under the criterion 70:30 (male:female), **(C)** PA loci under the criterion 80:20 (male:female), **(D)** SNP loci under the criterion 30:70 (male:female), **(E)** PA loci under the criterion 30:70 (male:female), **(F)** SNP loci under the criterion 20:80 (male:female) and **(G)** PA loci under the criterion 20:80 (male:female).

**FIGURE 2:**
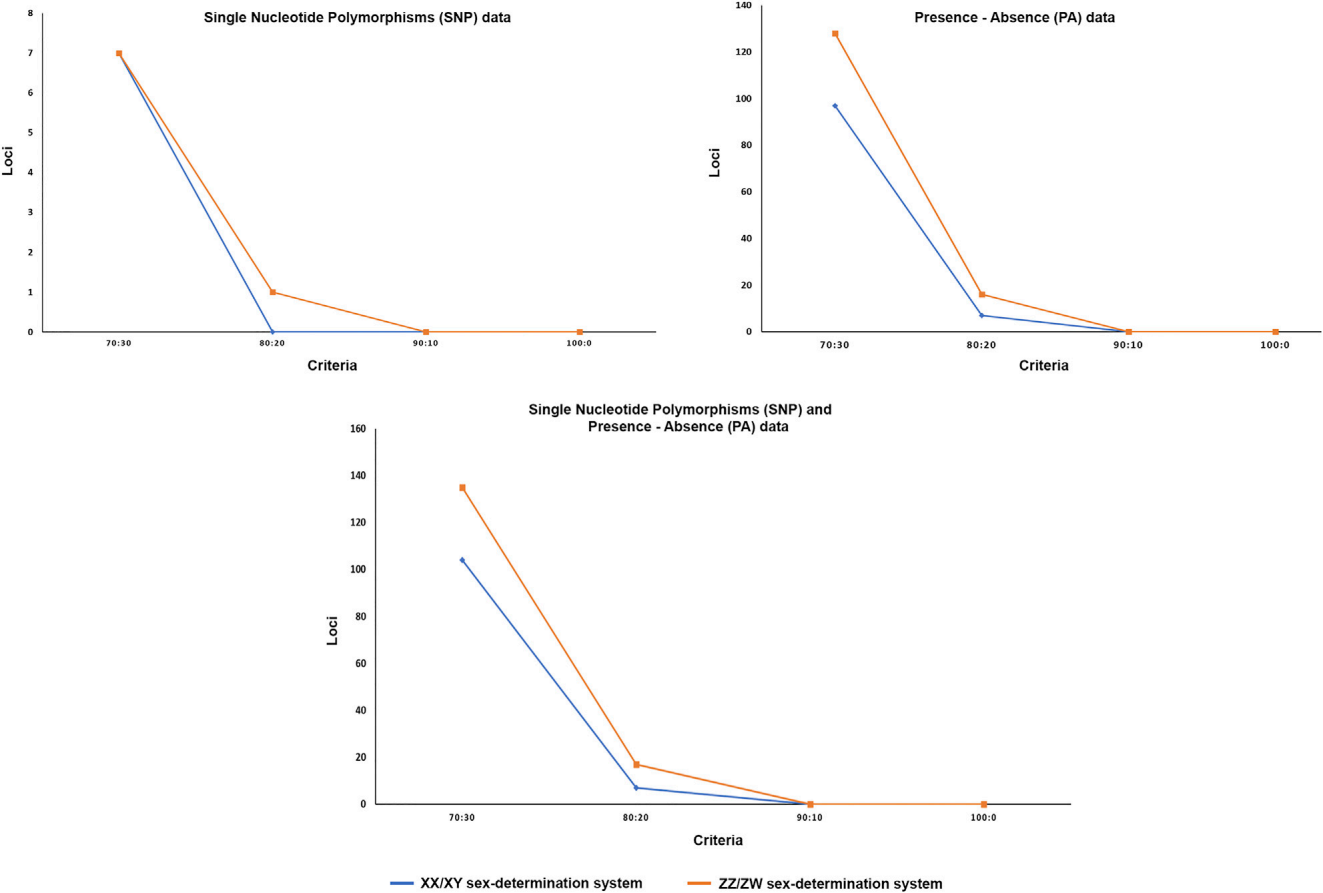
Graph showing number of loci for different hypotheses of sex determination systems after filtering with different criteria. *X*-axis indicates the number of loci and *Y*-axis shows the filter criteria.

The DArTseq methodology identified 50,348 SNP loci and 51,294 PA loci. Polymorphism information content values ranged from 0 to 0.50 for all loci, suggesting that the overall distribution of *PIC* values was asymmetrical and skewed toward higher values. Several SNP and PA loci were selected with a varying set of criteria (Table 1; Figure 1 and Figure 2) and compared to determine the existence of XX/XY or ZZ/ZW SDS in the hybrid. In the case of ZZ/ZW type, filtering using the criterion of 30:70 male:female showed in seven SNP loci and 128 PA loci as moderately female-linked (Supplementary Table S2, Figure 2). Hamming distances between male and female individuals using the moderately sex-linked SNP and PA loci indicated within-sex distances of 0.370 ± 0.032 in males and 0.382 ± 0.026 in females for SNP loci, and 0.613 ± 0.011 in males and 0.397 ± 0.019 in females for PA loci. Between-sex distances were 0.669 ± 0.019 for SNP loci and 0.712 ± 0.008 for PA loci. The CATT results showed a statistically significant association with the phenotypic sex for seven SNP loci (χ^2^ = 5.231–16.425, *p* < .022) and 128 PA loci (*χ* ^2^ = 4.320–13.715, *p* < .037) (Table 1; Supplementary Table S2, Figure 1). The criterion of 20:80 male:female involved the designation of one SNP locus and sixteen PA loci as moderately female-linked (Supplementary Table S2, Figure 2). Proportional pairwise Hamming distances between males and females using the moderately sex-linked SNP and PA loci (under the null exclusive model) showed within-sex distances of 0.133 ± 0.033 in males and 0.343 ± 0.047 in females for SNP loci, and 0.603 ± 0.017 in males and 0.338 ± 0.018 in females for PA loci. Between-sex distances were 0.760 ± 0.029 for SNP loci and 0.745 ± 0.009 for PA loci. The CATT results verified a significant association with phenotypic sex for one SNP locus (*χ* ^2^ = 16.425, *p* < 0.000) and 16 PA loci (χ^2^ = 6.250–13.715, *p* < 0.012) (Table 1; Supplementary Table S2, Figure 1).

**TABLE 1:** DArT analysis for 15 male and 15 female individuals of hybrid catfish (*Clarias gariepinus*, Burchell, 1822 x *Clarias macrocephalus*, Günther, 1864).

ZZ/ZW sex-determination type	30:70 male:female		20:80 male:female		10:90 male:female		0:100 male:female	
	SNP^a^	PA^b^	SNP^a^	PA^b^	SNP^a^	PA^b^	SNP^a^	PA^b^
Total number of DArT analyses	50,348	51,294	50,348	51,294				
Sex-linked loci	7	128	1	16	—	—	—	—
Overall mean distance between males and females	0.669 ± 0.019	0.712 ± 0.008	0.760 ± 0.029	0.745 ± 0.009	—	—	—	—
Overall mean distance within females	0.382 ± 0.026	0.397 ± 0.019	0.343 ± 0.047	0.338 ± 0.018	—	—	—	—
Overall mean distance within males	0.370 ± 0.032	0.613 ± 0.011	0.133 ± 0.033	0.603 ± 0.017	—	—	—	—
CATT test	*χ*2 = 5.231–16.425 *p* < .022	*χ*2 = 4.320–13.715 *p* < .037	*χ*2 = 16.425 *p* < .000	*χ*2 = 6.250–13.715 *p* < .012	—	—	—	—

XX/XY sex-determination type	70:30 male:female		80:20 male:female		90:10 male:female		100:0 male:female	
	SNP^a^	PA^b^	SNP^a^	PA^b^	SNP^a^	PA^b^	SNP^a^	PA^b^
Sex-linked loci	7	97	—	7	—	—	—	—
Overall mean distance between males and females	0.680 ± 0.014	0.719 ± 0.018	—	0.778 ± 0.020	—	—	—	—
Overall mean distance within females	0.457 ± 0.021	0.517 ± 0.033	—	0.539 ± 0.038	—	—	—	—
Overall mean distance within males	0.367 ± 0.02	0.403 ± 0.022	—	0.318 ± 0.022	—	—	—	—
CATT test	*χ*2 = 6.533–10.995 *p* < .001	*χ*2 = 4.572–16.250 *p* < .032	—	*χ*2 = 9.458–16.250 *p* < .002	—	—	—	—

a SNP, Single-nucleotide polymorphic loci.

b PA, Restriction fragment presence/absence loci.

**FIGURE 3:**
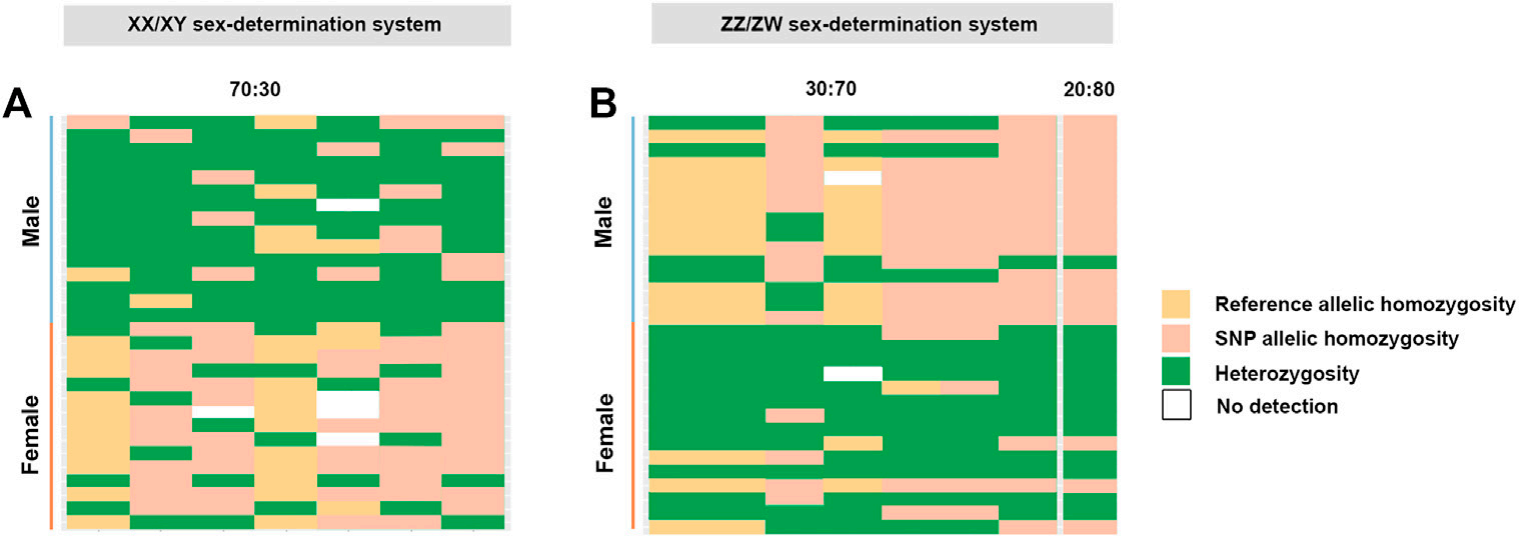
**(A)** Index of seven sex-linked loci filtered under the criterion 70:30 (male:female) (XX/XY sex-determination system) and **(B)** index of seven sex-linked loci filtered under the criteria 30:70 and index of one sex-linked loci filtered under the criteria 20:80 (male:female) (ZZ/ZW sex-determination system). Plots were generated using the “glPolt” function in the R package dartR (Gruber and Georges, 2021). Yellow shading indicates reference allelic homozygosity, green is indicative of heterozygosity and pink indicates SNP allelic homozygosity.

For the XX/XY determination system, filtering using the criterion of 70:30 male:female yielded seven SNP loci and 97 PA loci that were male-linked (Supplementary Table S3, Figure 2). Hamming distances between male and female bighead catfish using sex-linked SNP and PA loci (under the null exclusive model) showed lower within-sex distances of 0.367 ± 0.02 in males and 0.457 ± 0.021 in females for SNP loci, and 0.403 ± 0.022 in males and 0.517 ± 0.033 in females for PA loci. Between-sex distances of 0.680 ± 0.014 for SNP loci and 0.719 ± 0.018 for PA loci were observed. CATT results verified the significance of associations, with phenotypic sex for seven SNP loci (*χ* ^2^ = 6.533–10.995, *p* < .001) and 97 PA loci (*χ* ^2^ = 4.572–16.250, *p* < .032) (Table 1; Supplementary Table S3, Figure 1). No SNP loci and seven PA loci were associated with males based on the criterion of 80:20 male:female (Supplementary Table S3, Figure 2). Hamming distances between male and female hybrid catfish using sex-linked PA loci showed lower within-sex distances 0.318 ± 0.022 in males and 0.539 ± 0.038 in females for PA loci. Between-sex distances of 0.778 ± 0.020 for PA loci were observed. CATT results verified significant associations with phenotypic sex for seven PA loci (*χ* ^2^ = 9.458–16.250, *p* < .002) (Table 1; Supplementary Table S3, Figure 1). A glPlot showed similarity between sexes in the sample group for both XX/XY and ZZ/ZW SDSs when considering moderately sex-linked loci (Figure 3). Female sex-linked loci and male sex-linked loci of hybrid catfish indicated sequence homology with vertebrate genomes, based on global BLAST analyses of NCBI databases (Supplementary Tables S2,3). Regarding the ZZ/ZW type, ten of the 135 SNP and PA loci were homologous to the putative genes. Moreover, two SNP loci and 18 PA loci demonstrated partial homology with transposable elements (TEs) (Supplementary Tables S4,5). For PA loci, chi-square tests showed that the 30:70 and 20:80 filtering criteria demonstrated insignificant differences in males (*χ* ^2^ = 5.388 × 10^–32^, *p* = 1) and females (*χ* ^2^ = 1.959 × 10^–33^, *p* = 1). For SNP loci, Kruskal–Wallis tests indicated that these filtering criteria produced insignificantly different percentages of heterozygosity in males (H = 2.860, *p* = .091) and females (H = 6.840, *p* = .009) (Supplementary Figure S1). Calculation of pairwise comparisons using the Nemenyi-tests with chi-square approximation for independent samples revealed that the 30:70 and 20:80 filtering criteria resulted in insignificant differences in heterozygosity compared with other filters for males (*p* = .143) and females (*p* = .571). By contrast, with regard to the XX/XY sex chromosome system, six of 104 SNP and PA loci were homologous to the putative genes. Two SNP loci and twenty-one PA loci showed partial homology with TEs (Supplementary Tables S6,7). Chi-square tests showed that the 70:30 and 80:20 filtering criteria indicated insignificant differences in males (*χ* ^2^ = 3.962 × 10^–32^, *p* = 1) and females (*χ* ^2^ = 2.518 × 10^–32^, *p* = 1) for PA loci. The hybrid sex-linked loci were searched to find the genome-wide SNP of their parents (*C. gariepinus* × *C. macrocephalus*) and detect the loci inherited from their parents. The genome-wide SNP of their parents (*C. gariepinus* × *C. macrocephalus*) were used from our previous study, as described by Nguyen et al. (2021a); Nguyen et al. (2021b). Twenty-six candidate sex-linked markers were found in bighead catfish and three candidate sex-linked markers were found in African catfish, while 75 of the total 84 candidate sex-linked loci of the bighead catfish were found in the hybrids (Supplementary Table S8).

Across a range of sample sizes and loci, 30 phenotypically sexed individuals are essential to minimize the probability of selecting less than one spurious sex-linked marker. The probability (*P*_*i*_) of a single locus exhibited a sex-linked pattern by chance was 9.31 × 10^–10^. For the full data set (no filtering), out of 101,642 loci (including SNP and PA loci), 9.463 × 10^–5^ were expected to spuriously show perfect sex-linked loci based on chance alone.

## Discussion

Although the SDS of the last common ancestor of clariid fishes is not known, the hybrid catfish in this study provided a unique opportunity to study the various SDSs that are present in clariid fishes. Most SDSs are XY or ZW homomorphic sex chromosomes (Nguyen et al., 2021a). Due to the lability of sex determination processes in teleosts, closely related species sometimes possess different SDSs (Kitano and Peichel, 2012). Both sex determination systems might have undergone collateral evolution, or a transformation might have occurred in the common ancestor, which separated the lineages. Here, the genome-wide SNP of their parent (*C. gariepinus* x *C. macrocephalus*) was used from our previous research, as described by Nguyen et al. (2021a); Nguyen et al. (2021b). Female bighead catfish can produce only X gametes, whereas male African catfish can produce possible X, Y, and Z gametes. If both systems are present in the African catfish population in Thailand, their sex-linked SNP loci can be independently transferred from the male parent to the hybrid, thereby resulting in the presence of different SNP profiles in each individual. In this study, 26 candidate sex-linked markers came from the bighead catfish and three candidate sex-linked markers came from the African catfish. Seventy-five of the total 84 candidate sex-linked loci of the bighead catfish were observed in the hybrids but they were not all sex-linked loci in the hybrid (Supplementary Tables S8). This suggests that several sex-determining loci or clusters remain in the parental African catfish. Developmental pathways that are downstream of the primary sex-determining genes are conserved among various organisms having vastly different SDSs (Ezaz et al., 2016). These results collectively suggest that the variation in the SDSs of the parents might result in PSD in the hybrid. Male F_1_ hybrids are sterile because of the influence of different numbers of diploid chromosomes in their parents or because of the presence of complex SDSs. Sex-linked genes are important to determine the incompatibility between hybrids and the presence of non-homologous sex chromosomes could indicate reproductive isolation and increase the probability of speciation (Qvarnström and Bailey, 2009). In our previous study, we have shown that all sex-linked loci in African catfish and bighead catfish were not found in the same linkage group (Nguyen et al., 2021a; Nguyen et al., 2021b). Therefore, we predict that African catfish exhibit PSD on different chromosomes. PSD results in the generation of multiple phenotypic or reproductive classes within one sex. In the African catfish, more than two sex types have been observed in diverse populations (Teugels et al., 1992; Eyo and Effiong, 2005; Nguyen et al., 2021a). The presence of multiple classes may promote fitness benefits as natural selection in the population, suggesting that PSD engenders an evolutionarily stable scenario. Moreover, there is a noticeable diversity in how PSD has evolved separately across teleosts (Sandra and Norma, 2010). Genetic conflicts may also markedly impact hybrid sex determination and gametogenesis, with outcomes as intersex or sterile individuals, respectively and this can build strong post-zygotic barriers. PSD systems are evolutionarily unstable, because of sex-specific natural selection; they have generally been evaluated as only a midway step in the evolution of SDSs (Bachtrog et al., 2014).

Several reasons for the emergence of differences in SDSs in two closely related species have been postulated. With no sex chromosomes in the last common ancestor, both systems developed independently or one lineage retained the ancestral sex chromosomes, while transition occurred in other lineages (Mank et al., 2006). Such transitions can occur on the same pair of sex chromosomes or involved as an autosome that then became a new sex chromosome (Franchini et al., 2018). Sex-determining gene(s) at the molecular level may change their mode of action from female to male determination. Alternatively, a novel sex-determining (SD) gene might have initiated SD turnover in the evolving lineage. This change in mode of action of the genes may occur in the lineage of clariid fish, where both XX/XY and ZZ/ZW systems are found (Nguyen et al., 2021a; Nguyen et al., 2021b). By contrast, the platyfish has XY system, which is an ancestral SDS. One X chromosome acquired a female sex-determination allele dominant to the Y chromosome. This resulted in WY individuals developing ovaries. Several species of cichlid fish from Lake Malawi exhibited both an XY locus and WZ locus on different chromosome pairs. With occurrence on the same individual, the W female determines the dominant locus and the selected individual consequently develops as female (Ser et al., 2010). Ultimately, the dominant locus determines the fate of the gonad in the system being examined. Modification of existing sex chromosomes leads to multifactorial mechanisms, creating a functional third sex chromosome at the same or different locus. In this study, the female hybrids were fertile and able to produce large numbers of backcross progeny (Na-Nakorn et al., 2004; Ezaz et al., 2016). This suggested that the systems remain partially compatible (if one supersedes the other). We determined seven SNP or PA markers for male-linked loci and 17 SNP or PA markers for female-linked loci, with the criterion of 80:20 male:female across all examined hybrid specimens. Comparison of sex-linked loci of hybrids and the parent species showed no tendency of inheritance. Many false-positive signals might be desirable from such specimens due to their diverse genetic backgrounds (Gamble et al., 2015). Current data exhibited the possibility of this approach. The probability that a single locus exhibited sex linkage pattern by chance was 9.31 × 10^–10^, and when the full data set (without filtering) of 101,642 loci (including SNP and PA loci) was considered, 9.463 × 10^–5^ were expected to spuriously indicate sex-linkage. Thus, identification of any erroneously sex-linked loci by chance seems unlikely. This suggests the possibility of the presence of novel sex-linked genetic loci as a consequence of combination polygenes. Combining independently evolved sex chromosomes could also stimulate new combinations of sexual characters (Tanaka et al., 2007). Sex determination in zebrafish and cichlids likely results from a combination of additive and epistatic interactions at many loci (Parnell and Streelman, 2013). Surprisingly, the PA35637844 female-linked locus of the hybrid in the present study showed homology with the *B4GALNT1* gene, and this locus was derived from a non-sex-linked locus found in the bighead catfish. Our gene ontology search in the Ensembl v103 database confirmed that the *B4GALNT1* gene encodes functions related to the spermatogenesis pathway and hatchability (Izumi et al., 2019). Hence, the PA35637844 locus might have a putative role in the sex determination of the hybrids. An allele that wins in one combination of sex determination loci genotypes may lose in another, as observed from sex-linked loci of the bighead catfish. However, the influence of PSD remains in hybrids and African catfish. These outcomes illustrate the difficulty in predicting the consequences of SDSs on the speciation process that has only been studied in some cases.

## Conclusion

Hybrid catfish is of considerable economic importance for aquaculture as they can improve productivity through hybrid vigor, produce sterile animals or transfer desirable traits. Our findings revealed that SDS type ZZ/ZW can co-exist with XX/XY types as PSD in the same individuals of hybrid catfish. These models can be used to study the evolution of gene networks and epistasis, thereby allowing us to investigate the developmental regulation of genes that are traditionally thought to be members of core sex signaling networks. Identifying additional sex-determining genes and their interactions can offer unique insight to infer the process of sexual development. However, our results need to be extrapolated to other catfish species to investigate the evolution of SDSs in clariid fish. Chromosome mapping using fluorescence *in situ* hybridization on sex-linked loci and high-quality whole genome assemblies should be performed in catfish to identify the positions of sex-determining genes/loci.

## Data Availability

The datasets presented in this study can be found in online repositories. The names of the repository/repositories and accession number(s) can be found below: https://datadryad.org/stash, 10.5061/dryad.prr4xgxj6.

## References

[B1] Abol-Munafi A. B. Liem P. T. Ambak M. A. Siraj S. S. (2006). Effects of Maturational Hormone Treatment on Spermatogenesis of Hybrid Catfish (*Clarias macrocephalus X C. gariepinus*). J. Sustain. Sci. Manag. 1, 24–31.

[B2] Bachtrog D. Mank J. E. Peichel C. L. Kirkpatrick M. Otto S. P. Ashman T.-L. (2014). Sex Determination: Why So many Ways of Doing it? Plos Biol. 12, e1001899. 10.1371/journal.pbio.1001899 24983465 PMC4077654

[B3] Bao W. Kojima K. K. Kohany O. (2015). Repbase Update, a Database of Repetitive Elements in Eukaryotic Genomes. Mobile DNA 6, 11. 10.1186/s13100-015-0041-9 26045719 PMC4455052

[B51] Burchell, W. J. (1822). Travels in the Interior of Southern Africa. London: Creative media partners LLC.

[B4] Chaivichoo P. Koonawootrittriron S. Chatchaiphan S. Srimai W. Na-Nakorn U. (2020). Genetic Components of Growth Traits of the Hybrid between ♂North African Catfish (*Clarias gariepinus* Burchell, 1822) and ♀bighead Catfish (C. macrocephalus Günther, 1864). Aquaculture 521, 735082. 10.1016/j.aquaculture.2020.735082

[B5] Eding E. Bouwmans A. Komen J. (1997). “Evidence for a XX/XY Sex Determining Mechanism in the African Catfish *Clarias gariepinus* ,” in Presentation at the Sixth International Symposium on Genetics in Aquaculture, Stirling, Scotland.

[B6] Esmaeili H. R. Sayyadzadeh G. Amini Chermahini M. (2017). Sexual Dimorphism in Two Catfish Species, *Mystus Pelusius* (Solander, 1794) and *Glyptothorax Silviae* Coad, 1981 (Teleostei: Siluriformes). Turk. J. Zool. 41, 144–149. 10.3906/zoo-1509-22

[B7] Eyo J. Effiong J. (2005). Cytogenetic Variations in *Clarias* Species (Clariidae: Surulifromis) of the Anambra River Using Leucocytes Culture Techniques. Anim. Res. Int. 2, 275–286. 10.4314/ari.v2i1.40852

[B8] Ezaz T. Srikulnath K. Graves J. A. M. (2016). Origin of Amniote Sex Chromosomes: an Ancestral Super-sex Chromosome, or Common Requirements? Jhered 108, 94–105. 10.1093/jhered/esw053 27634536

[B9] Franchini P. Jones J. C. Xiong P. Kneitz S. Gompert Z. Warren W. C. (2018). Long-term Experimental Hybridisation Results in the Evolution of a New Sex Chromosome in Swordtail Fish. Nat. Commun. 9, 5136. 10.1038/s41467-018-07648-2 30510159 PMC6277394

[B10] Gamble T. Coryell J. Ezaz T. Lynch J. Scantlebury D. P. Zarkower D. (2015). Restriction Site-Associated DNA Sequencing (RAD-Seq) Reveals an Extraordinary Number of Transitions Among Gecko Sex-Determining Systems. Mol. Biol. Evol. 32, 1296–1309. 10.1093/molbev/msv023 25657328

[B11] Gammerdinger W. J. Kocher T. D. (2018). Unusual Diversity of Sex Chromosomes in African Cichlid Fishes. Genes 9, 480. 10.3390/genes9100480 PMC6210639 30287777

[B12] Gruber B. Georges A. (2021). DartR: Importing and Analysing SNP and Silicodart Data Generated by Genome-wide Restriction Fragment Analysis. Available at: https://cran.r-project.org/web/packages/dartR/index.html (Accessed May 28, 2021).

[B45] Günther A. (1866). Catalogue of the Physostomi, containing the families Salmonidae, Percopsidae, Galaxidae, Mormyridae, Gymnarchidae, Esocidae, Umbridae, Scombresocidae, Cyprinodontidae, in the collection of the British museum. London: British Museum.

[B47] Hamilton F. (1822). Account of the Fishes Found in the River Ganges and its Branches. London: Edinburgh.

[B13] Izumi H. Gen K. Lokman P. M. Hagihara S. Horiuchi M. Tanaka T. (2019). Maternal Transcripts in Good and Poor Quality Eggs from Japanese Eel, Anguilla Japonica -their Identification by Large‐scale Quantitative Analysis. Mol. Reprod. Dev. 86, 1846–1864. 10.1002/mrd.23273 31544986

[B14] Jaccoud D. Peng K. Feinstein D. Kilian A. (2001). Diversity Arrays: a Solid State Technology for Sequence Information Independent Genotyping. Nucleic Acids Res. 29, e25. 10.1093/nar/29.4.e25 11160945 PMC29632

[B15] Kilian A. Wenzl P. Huttner E. Carling J. Xia L. Blois H. (2012). Diversity Arrays Technology: a Generic Genome Profiling Technology on Open Platforms. Methods Mol. Biol. 888, 67–89. 10.1007/978-1-61779-870-2_5 22665276

[B16] Kitano J. Peichel C. L. (2012). Turnover of Sex Chromosomes and Speciation in Fishes. Environ. Biol. Fish. 94, 549–558. 10.1007/s10641-011-9853-8 PMC4459657 26069393

[B17] Koomgun T. Laopichienpong N. Singchat W. Panthum T. Phatcharakullawarawat R. Kraichak E. (2020). Genome Complexity Reduction High-Throughput Genome Sequencing of green iguana (*Iguana iguana*) Reveal a Paradigm Shift in Understanding Sex-Chromosomal Linkages on Homomorphic X and Y Sex Chromosomes. Front. Genet. 11, 556267. 10.3389/fgene.2020.556267 33193634 PMC7606854

[B18] Lambert M. R. Skelly D. K. Ezaz T. (2016). Sex-linked Markers in the North American green Frog (*Rana clamitans*) Developed Using DArTseq Provide Early Insight into Sex Chromosome Evolution. BMC Genom. 17, 844. 10.1186/s12864-016-3209-x PMC5084323 27793086

[B19] Laopichienpong N. Kraichak E. Singchat W. Sillapaprayoon S. Muangmai N. Suntrarachun S. (2021). Genome-wide SNP Analysis of Siamese Cobra (*Naja Kaouthia*) Reveals the Molecular Basis of Transitions between Z and W Sex Chromosomes and Supports the Presence of an Ancestral Super-sex Chromosome in Amniotes. Genomics 113, 624–636. 10.1016/j.ygeno.2020.09.058 33002626

[B46] Linnaeus C. (1978). Systema Naturae per Regna Tria Naturae, Secundum Classes, Ordines, Genera, Species, Cum Characteribus, Differentiis, Synonymis, Locis, tenth ed. Sweden: Laurentii Salvii.

[B20] Mank J. E. Promislow D. E. L. Avise J. C. (2006). Evolution of Alternative Sex-Determining Mechanisms in Teleost Fishes. Biol. J. Linn. Soc. 87, 83–93. 10.1111/j.1095-8312.2006.00558.x

[B21] Na-Nakorn U. (1995). “Comparison of Cold and Heat Shock to Induce Diploid Gynogenesisin Thai Walking Catfish (*Clarias macrocephalus*),” in Proceedings of the thirty-third Kasetsart university annual conference, Bangkok, 15 October,1995

[B22] Na-Nakorn U. Rangsin W. Boon-ngam J. (2004). Allotriploidy Increases Sterility in the Hybrid between *Clarias macrocephalus* and *Clarias gariepinus* . Aquaculture 237, 73–88. 10.1016/j.aquaculture.2004.02.032

[B23] Nguyen D. H. M. Panthum T. Ponjarat J. Laopichienpong N. Kraichak E. Singchat W. (2021a). An Investigation of ZZ/ZW and XX/XY Sex Determination Systems in North African Catfish (*Clarias gariepinus*, Burchell, 1822). Front. Genet. 11, 562856. 10.3389/fgene.2020.562856 33584785 PMC7874028

[B24] Nguyen D. H. M. Ponjarat J. Laopichienpong N. Kraichak E. Panthum T. Singchat W. (2021b). Genome-wide SNP Analysis Suggests Male Heterogamety in Bighead Catfish (*Clarias macrocephalus*, Günther, 1864). Aquaculture 543, 737005. 10.1016/j.aquaculture.2021.737005

[B25] Pandey N. Lakra W. S. (1997). Evidence of Female Heterogamety, B-Chromosome and Natural Tetraploidy in the Asian Catfish, *Clarias batrachus,* Used in Aquaculture. Aquaculture 149, 31–37. 10.1016/S0044-8486(96)01427-5

[B26] Panthum T. Singchat W. Laopichienpong N. Ahmad S. F. Kraichak E. Duengkae P. (2021). Genome-wide SNP Analysis of Male and Female rice Field Frogs, *Hoplobatrachus rugulosus*, Supports a Non-genetic Sex Determination System. Diversity 13, 501. 10.3390/d13100501

[B27] Parnell N. F. Streelman J. T. (2013). Genetic Interactions Controlling Sex and Color Establish the Potential for Sexual Conflict in Lake Malawi Cichlid Fishes. Heredity 110, 239–246. 10.1038/hdy.2012.73 23092997 PMC3668650

[B28] Pennell M. W. Mank J. E. Peichel C. L. (2018). Transitions in Sex Determination and Sex Chromosomes across Vertebrate Species. Mol. Ecol. 27, 3950–3963. 10.1111/mec.14540 29451715 PMC6095824

[B29] Ponjarat J. Singchat W. Monkheang P. Suntronpong A. Tawichasri P. Sillapaprayoon S. (2019). Evidence of Dramatic Sterility in F1 Male Hybrid Catfish [male *Clarias gariepinus* (Burchell, 1822) × Female *C. macrocephalus* (Günther, 1864)] Resulting from the Failure of Homologous Chromosome Pairing in Meiosis I. Aquaculture 505, 84–91. 10.1016/j.aquaculture.2019.02.035

[B30] Qvarnström A. Bailey R. I. (2009). Speciation through Evolution of Sex-Linked Genes. Heredity 102, 4–15. 10.1038/hdy.2008.93 18781167

[B48] Rafinesque C. S. (1818). American Monthly Magazine and Critical Review. Philadelphia: Academy of natural sciences of Philadelphi.

[B31] R Core Team (2021). R: *A Language And Environment for Statistical Computing* . Vienna: R foundation for statistical computing.

[B32] Ren R. Ray R. Li P. Xu J. Zhang M. Liu G. (2015). Construction of a High-Density DArTseq SNP-Based Genetic Map and Identification of Genomic Regions with Segregation Distortion in a Genetic Population Derived from a Cross between Feral and Cultivated-type Watermelon. Mol. Genet. Genomics 290, 1457–1470. 10.1007/s00438-015-0997-7 25702268

[B33] Roberts N. B. Juntti S. A. Coyle K. P. Dumont B. L. Stanley M. K. Ryan A. Q. (2016). Polygenic Sex Determination in the Cichlid Fish *Astatotilapia burtoni* . BMC Genom. 17, 835. 10.1186/s12864-016-3177-1 PMC5080751 27784286

[B34] Sandra G.-E. Norma M.-M. (2010). Sexual Determination and Differentiation in Teleost Fish. Rev. Fish. Biol. Fish. 20, 101–121. 10.1007/s11160-009-9123-4

[B35] Ser J. R. Roberts R. B. Kocher T. D. (2010). Multiple Interacting Loci Control Sex Determination in Lake Malawi Cichlid Fish. Evolution 64, 486–501. 10.1111/j.1558-5646.2009.00871.x 19863587 PMC3176681

[B36] Sopniewski J. Shams F. Scheele B. C. Kefford B. J. Ezaz T. (2019). Identifying Sex-Linked Markers in *Litoria aurea*: a Novel Approach to Understanding Sex Chromosome Evolution in an Amphibian. Sci. Rep. 9, 1659. 10.1038/s41598-019-52970-4 31719585 PMC6851140

[B37] Supikamolseni A. Ngaoburanawit N. Sumontha M. Chanhome L. Suntrarachun S. Peyachoknagul S. (2015). Molecular Barcoding of Venomous Snakes and Species-specific Multiplex PCR Assay to Identify Snake Groups for Which Antivenom Is Available in Thailand. Genet. Mol. Res. 14, 13981–13997. 10.4238/2015.october.29.18 26535713

[B38] Suprapto R. AlimudddinNuryati A. NuryatiMarnis S. Imron I. Marnis H. Iswanto B. (2017). MHC-II Marker Potential Linked to Motile Aeromonad Septicaemia Disease Resistance in African Catfish (*Clarias gariepinus*). Indonesian Aquacult. J. 12, 21–28. 10.15578/iaj.12.1.2017.21-28

[B39] Sven K. Klaus R. (2019). HapEstXXR: Multi-Locus Stepwise Regression. Available at: https://rdrr.io/cran/HapEstXXR/man/coding.baseline. allele.html (Accessed May 12, 2021).

[B40] Takeda H. (2008). Draft Genome of the Medaka Fish: a Comprehensive Resource for Medaka Developmental Genetics and Vertebrate Evolutionary Biology. Dev. Growth Differ. 50, S157–S166. 10.1111/j.1440-169X.2008.00992.x 18430160

[B41] Tanaka K. Takehana Y. Naruse K. Hamaguchi S. Sakaizumi M. (2007). Evidence for Different Origins of Sex Chromosomes in Closely Related *Oryzias* Fishes: Substitution of the Master Sex-Determining Gene. Genetics 177, 2075–2081. 10.1534/genetics.107.075598 17947439 PMC2219477

[B49] Temminck C. J. Schlegel H. (1846). Pisces in Siebold's Fauna Japonica. Batavia: Lugduni Batavorum.

[B50] Temminck C. J. Schlegel H. (1850). Fauna Japonica, Sive, Descriptio Animalium, Quae in Itinere per Japoniam, Jussu et Auspiciis, Superiorum, qui Summum in India Batava Imperium Tenent, Suscepto, Annis 1823–1830. Netherlands: Lugduni Batavorum, Apud Auctorem.

[B42] Teugels G. G. Ozouf-costz C. Legendre M. Parrent M. (1992). A Karyological Analysis of the Artificial Hybridization between *Clarias gariepinus* (Burchell, 1822) and *Heterobranchus longifilis* Valenciennes, 1840 (Pisces; Clariidae). J. Fish. Biol. 40, 81–86. 10.1111/j.1095-8649.1992.tb02555.x

[B43] Volff J.-N. Schartl M. (2001). Variability of Genetic Sex Determination in Poeciliid Fishes. Genetica 111, 101–110. 10.1023/a:1013795415808 11841158

[B44] Warren W. C. Hillier L. W. Tomlinson C. Minx P. Kremitzki M. Graves T. (2017). A New Chicken Genome Assembly Provides Insight into Avian Genome Structure. G3 (Bethesda) 7, 109–117. 10.1534/g3.116.035923 27852011 PMC5217101

