# Supplementary figures and images for: Genome-Wide SNP Analysis of Hybrid Clariid Fish Reflects the Existence of Polygenic Sex-Determination in the Lineage
Source: Front Genet. 2022 Feb 3;13:789573. doi: 10.3389/fgene.2022.789573 (PMC8851383; doi:10.3389/fgene.2022.789573)

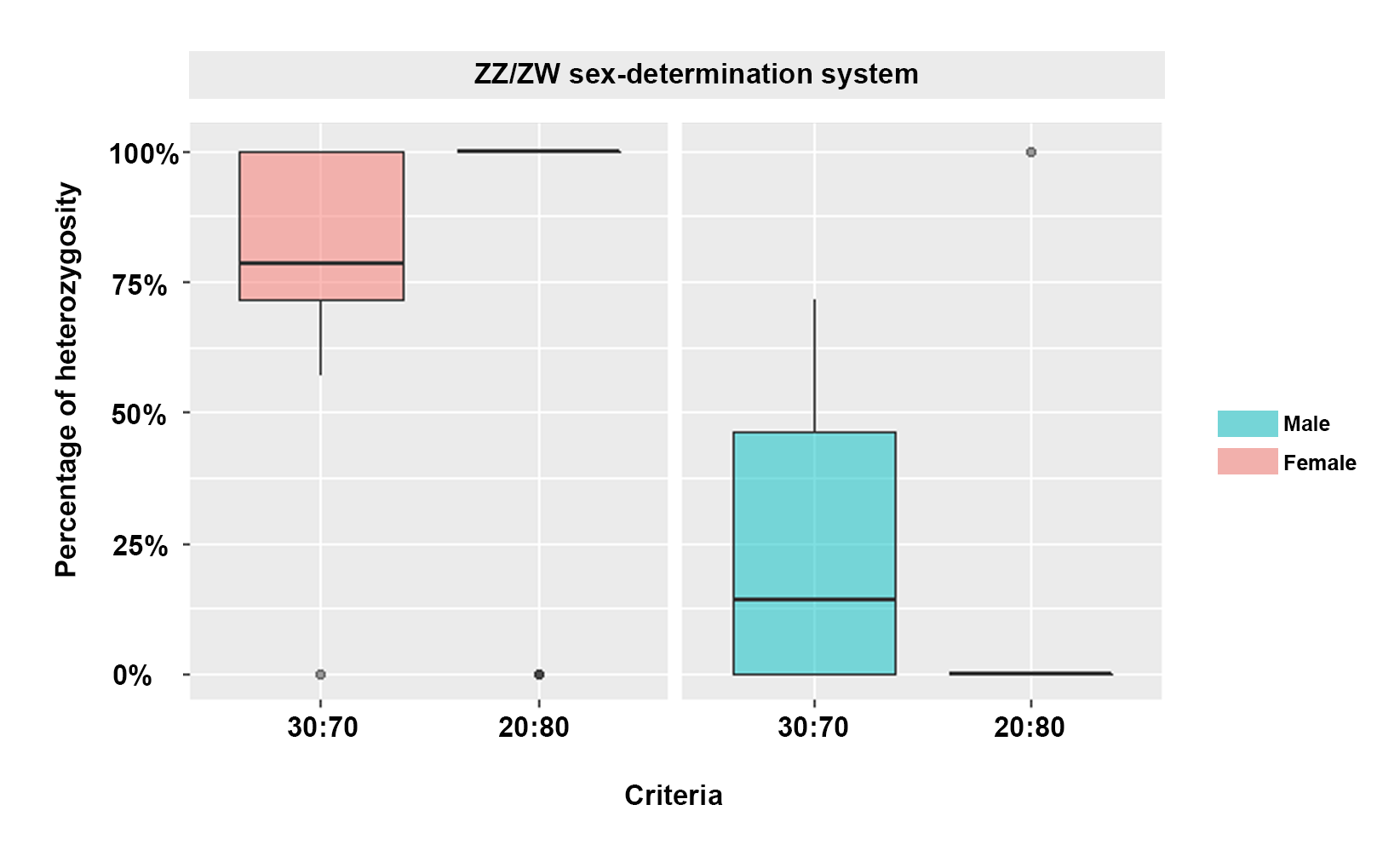

Supplement: Supplementary file 1 [file Image1.TIF]
