# Supplementary material for: Genome-Wide SNP Analysis of Hybrid Clariid Fish Reflects the Existence of Polygenic Sex-Determination in the Lineage
Source: Front Genet. 2022 Feb 3;13:789573. doi: 10.3389/fgene.2022.789573 (PMC8851383; doi:10.3389/fgene.2022.789573)
Supplement: Supplementary file 2 [file DataSheet1.docx]

**Supplementary Table S1** **|** Basic local alignment search tool (BLAST) search was performed to investigate the homologies of sex-linked SNP/PA loci against a selection of available teleost fish genomes as Japanese rice fish (*Oryzias latipes*, Temminck and Schlegel, 1846), zebrafish (*Danio rerio*, Hamilton, 1822), Japanese pufferfish (*Takifugu rubripes*, Temminck and Schlegel, 1850), channel catfish (*Ictalurus punctatus*, Rafinesque, 1818), and an amniote reference genome (chicken, *Gallus gallus*, Linnaeus, 1758).

| Species | Accession number | Genome size | Reference |
| --- | --- | --- | --- |
| *Oryzias latipes* | GCA_002234675.1 | 746.737 Mb | Kasahara et al. (2007) |
| *Danio rerio* | GCA_000002035.4 | 1408.43 Mb | Broughton et al. (2001) |
| *Takifugu rubripes* | GCA_901000725.2 | 384.127 Mb | Elmerot et al. (2002) |
| *Ictalurus punctatus* | GCF_001660625.1 | 910.132 Mb | Liu et al. (2016) |
| *Gallus gallus* | GCA_000002315.5 | 1037.17 Mb | International Chicken Genome Sequencing Consortium (2004) |

Broughton, R. E., Milam, J. E., and Roe, B. A. (2001). The complete sequence of the zebrafish (*Danio rerio*) mitochondrial genome and evolutionary patterns in vertebrate mitochondrial DNA. *Genome Res*. 11, 1958–1967. doi: 10.1101/gr.156801

Elmerot, C., Amason, U., Gojobori, T., and Janke, A. (2002). The mitochondrial genome of the pufferfish, *Fugu rubripes*, and ordinal teleost relationships. *Gene* *295*, 163–172. doi: 10.1016/s0378-1119(02)00688-1 33

International chicken genome sequencing consortium, (2004). Sequence and comparative analysis of the chicken genome provide unique perspectives on vertebrate evolution. *Nature* 432, 695–716, doi: 10.1038/nature03154

Kasahara, M., Naruse, K., Sasaki, S., Nakatani, Y., Qu, W., Ahsan, B., et al. (2007). The medaka draft genome and insights into vertebrate genome evolution. *Nature* 447, 714–719. doi: 10.1038/nature05846

Liu, Z., Liu, S., Yao, J., Bao, L., Zhang, J., Li, Y., et al. (2016). The channel catfish genome sequence provides insights into the evolution of scale formation in teleosts. *Nat*. *Commun*. 2: 11757. doi: 10.1038/ncomms11757.

**Supplementary Table S2 |** Chromosomal locations for single-nucleotide polymorphic and restriction fragment presence/absence loci of hybrid catfish (*Clarias gariepinus*, Burchell, 1822 x *Clarias macrocephalus*, Günther, 1864) from a BLAST homology search of the genomes of Japanese rice fish (*Oryzias latipes*, Temminck and Schlegel, 1846), zebrafish (*Danio rerio*, Hamilton, 1822), Japanese puffer (*Takifugu rubripes*, Temminck and Schlegel, 1850), channel catfish (*Ictalurus punctatus*, Rafinesque, 1818) and chicken (*Gallus gallus*, Linnaeus, 1758) (30:70, male:female; ZZ/ZW sex-determination type).

| Locus id | *Oryzias latipes* | *Danio rerio* | *Takifugu rubripes* | *Ictalurus punctatus* | *Gallus gallus* |
| --- | --- | --- | --- | --- | --- |
| PA55505684^a^ | - | - | - | 5 | - |
| PA40046663^b^ | - | - | - | 3 | - |
| PA55487337^a^ | - | - | - | 16 | - |
| PA55476486^a^ | - | - | - | 2 |  |
| PA35640437^a^ | - | - | - | 23 | - |
| PA55536456^b^ | 18 | - | 8 | 1 | - |
| PA55496212^b^ | - | - | - | 3 | - |
| PA55535477^b^ | - | - | - | 8 | - |
| PA55524427^b^ | - | - | - | 5 | - |
| PA55516444^b^ | - | - | - | 12 | - |
| PA55528515^a^ | 19 | - | - | 13 | - |
| PA55510013^a^ | 7 | 3 | - | 11 | - |
| PA55519860^a^ | - | - | - | 17 | - |
| PA55503883^a^ | 3 | 3 | - | 2 | 5 |
| PA100020705^a^ | 21 | - | - | - | - |
| PA55479687^a^ | 6 | 12 | - | 9 | 3 |
| PA55534802^a^ | 11 | - | - | 1 | - |
| PA35641017^a^ | 22 | 25 | - | 23 | - |
| PA55474895^b^ | - | 23 | - | - | - |
| PA55529448^a^ | - | 22 | - | 8 | - |
| PA55513702^a^ | - | - | - | 13 | - |
| PA35626878^a^ | - | - | - | 29 | - |
| PA40060478^a^ | - | 9 | - | 6 | - |
| PA35641360^a^ | - | - | - | 3 | - |
| PA55508371^a^ | - | - | - | 23 | ^-^ |
| PA55481919^a^ | - | 7 | 4 | 2 | - |
| PA55502629^a^ | - | - | - | 27 | - |
| PA55487558^a^ | - | 21 | - | 18 | - |
| PA55483125^a^ | - | - | - | 23 | - |
| PA55474915^a^ | - | - | - | 21 | - |
| PA35624597^a^ | - | 20 | - | 17 | - |
| PA55519585^a^ | - | 14 | - | 16 | - |
| PA100021328^a^ | - | - | - | 17 | - |
| PA40052848^a^ | - | 3 | - | 18 | 7 |
| PA55508298^a^ | - | 16 | - | - | - |
| PA55484090^a^ | - | 10 | - | 2 | - |
| PA35624997^a^ | - | 14 | - | 19 | - |
| PA35637844^a^ | - | 25 | - | 14 | - |
| PA55511323^a^ | - | - | - | 13 | - |
| PA55469998^a^ | - | - | - | 6 | - |
| PA55511114^a^ | - | - | - | 12 | - |
| PA55530629^a^ | - | - | - | 13 | - |
| PA55529973^a^ | - | - | - | 3 | - |
| PA55476711^a^ | - | - | - | 21 | - |
| PA100002504^a^ | - | - | - | 18 | - |
| PA55479677^a^ | - | - | - | 12 | - |
| PA100046160^a^ | - | - | - | 23 | - |
| PA55469848^a^ | - | - | - | 8 | - |
| PA55475486^a^ | - | - | - | 3 | - |
| PA55504947^a^ | - | - | - | 8 | - |
| PA55500305^a^ | - | - | - | 14 | - |
| PA55516442^a^ | - | - | - | 13 | - |
| PA55495523^a^ | - | - | - | 28 | - |
| PA55501648^a^ | - | - | - | 4 | - |
| PA55488118^a^ | - | - | - | 4 | - |
| PA40058344^a^ | - | - | - | 23 | - |
| PA35627651^b^ | - | - | - | - | - |
| PA55495980^b^ | - | - | - | - | - |
| PA55507853^b^ | - | - | - | - | - |
| PA35643024^b^ | - | - | - | - | - |
| PA100057811^b^ | - | - | - | - | - |
| PA55503612^b^ | - | - | - | - | - |
| PA55525504^b^ | - | - | - | - | - |
| PA55468178^b^ | - | - | - | - | - |
| PA35627301^b^ | - | - | - | - | - |
| PA55467581^a^ | - | - | - | - | - |
| PA55518015^a^ | - | - | - | - | - |
| PA100055159^a^ | - | - | - | - | - |
| PA55468043^a^ | - | - | - | - | - |
| PA35636078^a^ | - | - | - | - | - |
| PA35640559^a^ | - | - | - | - | - |
| PA55499649^a^ | - | - | - | - | - |
| PA100014363^a^ | - | - | - | - | - |
| PA55527555^a^ | - | - | - | - | - |
| PA55484477^a^ | - | - | - | - | - |
| PA100030740^a^ | - | - | - | - | - |
| PA55499834^a^ | - | - | - | - | - |
| PA55507832^a^ | - | - | - | - | - |
| PA55524616^a^ | - | - | - | - | - |
| PA55504623^a^ | - | - | - | - | - |
| PA55502367^a^ | - | - | - | - | - |
| PA55478613^a^ | - | - | - | - | - |
| PA55486086^a^ | - | - | - | - | - |
| PA55529720^a^ | - | - | - | - | - |
| PA100004476^a^ | - | - | - | - | - |
| PA 35636016^a^ | - | - | - | - | - |
| PA55525009^a^ | - | - | - | - | - |
| PA 55479014^a^ | - | - | - | - | - |
| PA55525871^a^ | - | - | - | - | - |
| PA55518666^a^ | - | - | - | - | - |
| PA55529933^a^ | - | - | - | - | - |
| PA55520474^a^ | - | - | - | - | - |
| PA55509461^a^ | - | - | - | - | - |
| PA55517899^a^ | - | - | - | - | - |
| PA55532944^a^ | - | - | - | - | - |
| PA55522529^a^ | - | - | - | - | - |
| PA55522660^a^ | - | - | - | - | - |
| PA55499691^a^ | - | - | - | - | - |
| PA55493969^a^ | - | - | - | - | - |
| PA55496394^a^ | - | - | - | - | - |
| PA35638008^a^ | - | - | - | - | - |
| PA35639998^a^ | - | - | - | - | - |
| PA100000476^a^ | - | - | - | - | - |
| PA55525304^a^ | - | - | - | - | - |
| PA100000461^a^ | - | - | - | - | - |
| PA40060842^a^ | - | - | - | - | - |
| PA55522094^a^ | - | - | - | - | - |
| PA55531935^a^ | - | - | - | - | - |
| PA35642856^a^ | - | - | - | - | - |
| PA55501469^a^ | - | - | - | - | - |
| PA55509898^a^ | - | - | - | - | - |
| PA35639366^a^ | - | - | - | - | - |
| PA55512838^a^ | - | - | - | - | - |
| PA55508840^a^ | - | - | - | - | - |
| PA55479922^a^ | - | - | - | - | - |
| PA55519673^a^ | - | - | - | - | - |
| PA55477822^a^ | - | - | - | - | - |
| PA35644767^a^ | - | - | - | - | - |
| PA55516866^a^ | - | - | - | - | - |
| PA35644970^a^ | - | - | - | - | - |
| PA55478561^a^ | - | - | - | - | - |
| PA55504589^a^ | - | - | - | - | - |
| PA100001870^a^ | - | - | - | - | - |
| PA55499387^a^ | - | - | - | - | - |
| PA55529552^a^ | - | - | - | - | - |
| PA35638345^a^ | - | - | - | - | - |
| PA55514641^a^ | - | - | - | - | - |
| PA40048686^a^ | - | - | - | - | - |
| SNP100029825^a^ | - | - | - | - | - |
| SNP100031227^a^ | - | - | - | - | - |
| SNP40052530^a^ | - | - | - | 16 | - |
| SNP35642896^a^ | - | - | - | - | - |
| SNP55513876^a^ | - | - | - | 28 | - |
| SNP55489450^a^ | - | - | - | 8 | - |
| SNP55523064^b^ | - | - | - | 16 | - |

^a^ indicate 30:70 male:female without 20:80 male:female, and ^b^ indicate 20:80 male:female.

Burchell, W. J. (1822). *Travels in the Interior of Southern Africa*. London: Creative Media Partners LLC.

Günther, A. (1864). *Catalogue of Fishes in the British Museum London.* London: Natural History Museum Press.

**Supplementary Table S3 |** Chromosomal locations for single-nucleotide polymorphic and restriction fragment presence/absence loci of hybrid catfish (*Clarias gariepinus*, Burchell, 1822 x *Clarias macrocephalus*, Günther, 1864) from a BLAST homology search of the genomes of Japanese rice fish (*Oryzias latipes*, Temminck and Schlegel, 1846), zebrafish (*Danio rerio*, Hamilton, 1822), Japanese puffer (*Takifugu rubripes*, Temminck and Schlegel, 1850), channel catfish (*Ictalurus punctatus*, Rafinesque, 1818) and chicken (*Gallus gallus*, Linnaeus, 1758) (70:30, male:female; XX/XY sex-determination type).

| Locus id | *Oryzias latipes* | *Danio rerio* | *Takifugu rubripes* | *Ictalurus punctatus* | *Gallus gallus* |
| --- | --- | --- | --- | --- | --- |
| PA100047982^a^ | 6 | 22 | 8 | 19 | Z |
| PA55524816^a^ | 11 | - | 1 | - | - |
| PA35630234^b^ | - | - | - | 5 | - |
| PA55492443^b^ | - | - | - | 19 | - |
| PA100005297^a^ | - | - | - | 19 | - |
| PA55484322^a^ | 16 | - | 19 | 2 | - |
| PA55528570^a^ | 23 | - | - | - | - |
| PA55498173^a^ | 18 | - | - | 6 | - |
| PA55531700^a^ | - | 7 | - | 7 | - |
| PA100051674^a^ | - | 18 | 11 | 9 | - |
| PA55490785^a^ | - | 25 | - | - | - |
| PA55494593^a^ | - | 4 | - | 25 | - |
| PA55495872^a^ | - | - | - | 10 | - |
| PA55522914^a^ | 10 | 21 | 3 | 16 | - |
| PA55527404^a^ | - | 14 | - | - | 1 |
| PA55533797^a^ | - | 10 | - | 29 | - |
| PA100053786^a^ | - | 7 | - | 7 | - |
| PA100004818^a^ | - | 19 | - | 21 | - |
| PA100005234^a^ | - | 19 | - | - | - |
| PA35626399^a^ | - | 6 | - | 3 | - |
| PA100057746^a^ | 3 | 19 | 1 | 12 | - |
| PA55532208^a^ | - | 7 | 17 | 2 | - |
| PA100005162^a^ | - | 24 | 17 | 16 | - |
| PA55501166^a^ | - | 11 | - | 22 | - |
| PA100004820^a^ | - | 22 | - | 20 | - |
| PA55504535^a^ | - | 15 | - | - | - |
| PA55470656^b^ | - | - | - | 28 | - |
| PA100043843^a^ | - | - | - | 29 | - |
| PA55482614^a^ | - | - | - | 16 | - |
| PA55526856^a^ | - | - | - | 13 | - |
| PA55496004^a^ | - | - | - | 10 | - |
| PA55479719^a^ | - | - | - | 16 | - |
| PA100005082^a^ | - | - | - | 10 | - |
| PA100042814^a^ | - | - | - | 4 | - |
| PA100057295^a^ | - | - | - | 24 | - |
| PA55517469^a^ | - | - | - | 11 | - |
| PA100044562^a^ | - | - | - | 2 | - |
| PA55530372^a^ | - | - | - | 7 | - |
| PA55470050^a^ | - | - | - | 28 | - |
| PA35641359^a^ | - | - | - | 3 | - |
| PA100005251^a^ | - | - | - | 1 | - |
| PA100033944^a^ | - | - | - | 18 | - |
| PA55521148^a^ | - | - | - | 7 | - |
| PA100005043^a^ | - | - | - | 29 | - |
| PA55494250^b^ | - | - | - | - | - |
| PA55493618^b^ | - | - | - | - | - |
| PA100005396^b^ | - | - | - | - | - |
| PA 55473966^b^ | - | - | - | - | - |
| PA55470108^a^ | - | - | - | - | - |
| PA55535054^a^ | - | - | - | - | - |
| PA55469200^a^ | - | - | - | - | - |
| PA100048045^a^ | - | - | - | - | - |
| PA55511757^a^ | - | - | - | - | - |
| PA55488863^a^ | - | - | - | - | - |
| PA55510551^a^ | - | - | - | - | - |
| PA55502309^a^ | - | - | - | - | - |
| PA100004518^a^ | - | - | - | - | - |
| PA100005195^a^ | - | - | - | - | - |
| PA100006023^a^ | - | - | - | - | - |
| PA100025125^a^ | - | - | - | - | - |
| PA100004456^a^ | - | - | - | - | - |
| PA55471963^a^ | - | - | - | - | - |
| PA100022508^a^ | - | - | - | - | - |
| PA100005250^a^ | - | - | - | - | - |
| PA55534317^a^ | - | - | - | - | - |
| PA55517173^a^ | - | - | - | - | - |
| PA55473341^a^ | - | - | - | - | - |
| PA35627549^a^ | - | - | - | - | - |
| PA55497692^a^ | - | - | - | - | - |
| PA100006001^a^ | - | - | - | - | - |
| PA55521225^a^ | - | - | - | - | - |
| PA55509959^a^ | - | - | - | - | - |
| PA35624731^a^ | - | - | - | - | - |
| PA55495281^a^ | - | - | - | - | - |
| PA35630019^a^ | - | - | - | - | - |
| PA100004534^a^ | - | - | - | - | - |
| PA55510649^a^ | - | - | - | - | - |
| PA55530418^a^ | - | - | - | - | - |
| PA55471415^a^ | - | - | - | - | - |
| PA100005472^a^ | - | - | - | - | - |
| PA55476528^a^ | - | - | - | - | - |
| PA55496539^a^ | - | - | - | - | - |
| PA35629714^a^ | - | - | - | - | - |
| PA100024013^a^ | - | - | - | - | - |
| PA100005080^a^ | - | - | - | - | - |
| PA55503173^a^ | - | - | - | - | - |
| PA55499226^a^ | - | - | - | - | - |
| PA100004584^a^ | - | - | - | - | - |
| PA55486789^a^ | - | - | - | - | - |
| PA55522209^a^ | - | - | - | - | - |
| PA55513168^a^ | - | - | - | - | - |
| PA55532695^a^ | - | - | - | - | - |
| PA100055232^a^ | - | - | - | - | - |
| PA55477824^a^ | - | - | - | - | - |
| PA100004599^a^ | - | - | - | - | - |
| PA40059537^a^ | - | - | - | - | - |
| PA100025468^a^ | - | - | - | - | - |
| SNP35624822^a^ | - | - | - | - | - |
| SNP100030435^a^ | - | 5 | - | - | - |
| SNP35626133^a^ | - | 4 | - | - | - |
| SNP40053450^a^ | - | 15 | - | 17 | - |
| SNP55506555^a^ | - | - | - | - | - |
| SNP55486789^a^ | - | - | - | - | - |
| SNP55524029^a^ | - | - | - | 16 | - |

^a^ indicate 70:30 male:female without 80:20 male:female, and ^b^ indicate 80:20 male:female.

Burchell, W. J. (1822). *Travels in the Interior of Southern Africa*. London: Creative Media Partners LLC.

Günther, A. (1864). *Catalogue of Fishes in the British Museum London.* London: Natural History Museum Press.

**Supplementary Table S4** **|** Gene function and pathway for single-nucleotide polymorphic and restriction fragment presence/absence loci of hybrid catfish (*Clarias gariepinus*, Burchell, 1822 x *Clarias macrocephalus*, Günther, 1864) from a BLAST search of the genomes of Japanese rice fish (*Oryzias latipes*, Temminck and Schlegel, 1846), zebrafish (*Danio rerio*, Hamilton, 1822), Japanese puffer (*Takifugu rubripes*, Temminck and Schlegel, 1850), channel catfish (*Ictalurus punctatus*, Rafinesque, 1818) and chicken (*Gallus gallus*, Linnaeus, 1758) (30:70, male:female; ZZ/ZW sex-determination type).

| Locus id | Gene^1,2^ | E-values | Query coverage | Similarity | Product | Function | Component | Pathway | Reference |
| --- | --- | --- | --- | --- | --- | --- | --- | --- | --- |
| PA55487558 | *NUP188* | 2 x 10^-7^ | 73% | 86.27% | Nucleoporin NUP188 | Structural constituent of nuclear pore | Nuclear pore complex | Mitotic nuclear envelope reassembly, protein import into nucleus, regulation of glycolytic process | Muir et al. (2020) |
| PA55503883 | *fthl31* | 6 x 10^-13^ | 81% | 91.07% | Ferritin heavy polypeptide-like 17 | Ferric iron binding,  ferrous iron binding,  ferroxidase activity | Cytoplasm | Intracellular sequestering of iron ion, iron ion transport | Gaudet et al. (2011) |
| PA55487337 | *BACH1* | 2 x 10^-24^ | 100% | 95.65% | Transcription regulator protein BACH1 | DNA-binding transcription activator activity, RNA polymerase II-specific | Nucleus | DNA repair, negative regulation of transcription by RNA polymerase II | Tan et al. (2013) |
|  |  |  |  |  |  |  |  |  |  |
| PA55529448 | *CTNNBIP1* | 1 x 10^-9^ | 89% | 82.26% | Beta-catenin-interacting protein 1 | Armadillo repeat domain binding,  beta-catenin binding | Cytoplasm, nucleus | Anterior/posterior pattern specification,  branching involved in ureteric bud morphogenesis,  negative regulation of DNA binding | Graham et al. (2002) |
| PA55481919 | *SYNM* | 4 x 10^-9^ | 95% | 80.30% | Synemin | Intermediate filament binding,  structural constituent of cytoskeleton | Cytoskeleton | Fast-twitch skeletal muscle fibre contraction,  intermediate filament cytoskeleton organisation | Mizuno et al. (2001) |
| PA55479687 | *LARP4* | 0.001 | 76% | 79.25% | La-related protein 4 | mRNA 3'-UTR binding,  poly(A) binding,  RNA binding | Cytosol | cytoskeleton organisation,  positive regulation of translation, posttranscriptional regulation of gene expression | Bai et al. (2011) |
| PA55476711 | *TASOR* | 3 x 10^-4^ | 89% | 75.81% | Protein TASOR | Chromatin binding,  RNA binding | Nucleus | Anterior/posterior axis specification, embryo,  in utero embryonic development, mitotic spindle assembly | Tchasovnikarova et al. (2015) |
| PA55479677 | *CSNK2B* | 1 x 10^-8^ | 91% | 82.54% | Casein kinase II subunit beta | Chromatin binding,  identical protein binding, transcription factor binding | Cytosol, nucleus | Adiponectin-activated signalling pathway, cellular protein-containing complex assembly, mitotic chromosome condensation | Keller et al. (2001) |
| PA35637844 | *B4GALNT1* | 7 x 10^-6^ | 60% | 85.71% | Beta-1,4 N-acetylgalactosaminyltransferase 1 | (N-acetylneuraminyl)-galactosylglucosylceramide N-acetylgalactosaminyltransferase activity,  acetylgalactosaminyltransferase activity | Golgi apparatus membrane, Single-pass type II, membrane protein | Carbohydrate metabolic process,  ganglioside biosynthetic process, spermatogenesis | Nagata et al. (1992) |
| PA55501648 | *CYTH2* | 7 x 10^-6^ | 88% | 77.05% | Cytohesin-2 | guanyl-nucleotide exchange factor activity,  inositol 1,4,5 trisphosphate binding,  lipid binding | Cytoplasm, plasma membrane | actin cytoskeleton organisation,  endocytosis,  regulation of ARF protein signal transduction | Hofmann et al. (2007) |
|  |  |  |  |  |  |  |  |  |  |

^1^All genes show sequence similarity with a partial sequence of DArT loci of more than 60% from the NCBI database.

^2^All genes show an E-value with a partial sequence of DArT loci lower than 0.005 from the NCBI database.

Burchell, W. J. (1822). *Travels in the Interior of Southern Africa*. London: Creative Media Partners LLC.

Bai, S. W., Herrera-Abreu, M. T., Rohn, J. L., Racine, V., Tajadura, V., Suryavanshi, N., et al. (2011). Identification and characterization of a set of conserved and new regulators of cytoskeletal organization, cell morphology and migration. *BMC* *Biol*. 9: 54. doi: 10.1186/1741-7007-9-54

Gaudet, P., Livstone, M. S., Lewis, S. E., and Thomas, P. D. (2011). Phylogenetic-based propagation of functional annotations within the gene ontology consortium. *Brief. Bioinform*. 12, 449–462. [doi: 10.1093/bib/bbr042](https://doi.org/10.1093/bib/bbr042)

Günther, A. (1864). *Catalogue of Fishes in the British Museum London.* London: Natural History Museum Press.

Graham, T. A., Clements, W. K., Kimelman, D., and Xu, W. (2002). The crystal structure of the beta-catenin/ICAT complex reveals the inhibitory mechanism of ICAT. *Mol. Cell* 10, 563–571. doi: 10.1016/s1097-2765(02)00637-8

Hofmann, I., Thompson, A., Sanderson, C. M., and Munro, S. (2007). The Arl4 family of small G proteins can recruit the cytohesin Arf6 exchange factors to the plasma membrane. *Curr. Biol.* 17, 711–716. doi: 10.1016/j.cub.2007.03.007

Keller, D. M., Zeng, X., Wang, Y., Zhang, Q. H., Kapoor, M., Shu, H., et al. (2001). A DNA damage-induced p53 serine 392 kinase complex contains CK2, hSpt16, and SSRP1. *Mol*. *Cell* 7, 283–292. doi: 10.1016/s1097-2765(01)00176-9

Mizuno, Y., Thompson, T. G., Guyon, J. R., Lidov, H. G., Brosius, M., Imamura, M., et al. (2001). Desmuslin, an intermediate filament protein that interacts with alpha-dystrobrevin and desmin. *Proc. Natl. Acad. Sci. U.S.A* 98, 6156–6161. doi: 10.1073/pnas.111153298

Muir, A. M., Cohen, J. L., Sheppard, S. E., Guttipatti, P., Lo, T. Y., Weed, N., et al. (2020). Bi-allelic Loss-of-Function Variants in *NUP188* Cause a Recognizable Syndrome Characterized by Neurologic, Ocular, and Cardiac Abnormalities. *Am. J. Hum. Genet*. 106, 623–631. doi: 10.1016/j.ajhg.2020.03.009

Nagata, Y., Yamashiro, S., Yodoi, J., Lloyd, K. O., Shiku, H., and Furukawa, K. (1992). Expression cloning of beta 1,4 N-acetylgalactosaminyltransferase cDNAs that determine the expression of G_M2_ and G_D2_ gangliosides. *J. Biol. Chem*. 267, 12082–12089.

Tan, M. K., Lim, H. J., Bennett, E. J., Shi, Y., and Harper, J. W. (2013). Parallel SCF adaptor capture proteomics reveals a role for SCFFBXL17 in NRF2 activation via BACH1 repressor turnover. *Mol. Cell* 52, 9–24. doi: 10.1016/j.molcel.2013.08.018

Tchasovnikarova, I. A., Timms, R. T., Matheson, N. J., Wals, K., Antrobus, R., Göttgens, B., et al. (2015). Epigenetic silencing by the HUSH complex mediates position-effect variegation in human cells. *Science* 348, 1481–1485. doi: 10.1126/science.aaa7227

**Supplementary Table S5 |** Repeat searches for single-nucleotide polymorphic and restriction fragment presence/absence loci of hybrid catfish (*Clarias gariepinus*, Burchell, 1822 x *Clarias macrocephalus*, Günther, 1864) (ZZ/ZW sex-determination type).

| Repeat^1^ | Type | PA loci | SNP loci | Total |
| --- | --- | --- | --- | --- |
| 30:70, male:female | | | | |
| DNA transposon | IS3EU | 1 | - | 1 |
|  | Tc1/mariner | 6 | - | 6 |
|  | hAT | 3 | - | 3 |
| LTR retrotransposon | Gypsy | 5 | 2 | 7 |
|  | DIRS | 1 | - | 1 |
| Non-LTR retrotransposon |  | 1 | - | 1 |
|  | L2 | 1 | - | 1 |
| 20:80, male:female | | | | |
| DNA transposon | hAT | 1 | - | 1 |
| LTR retrotransposon | Gypsy | 1 | - | 1 |

^1^LTR, long terminal repeats.

Burchell, W. J. (1822). *Travels in the Interior of Southern Africa*. London: Creative Media Partners LLC.

Günther, A. (1864). *Catalogue of Fishes in the British Museum London.* London: Natural History Museum Press.

**Supplementary Table S6 |** Gene function and pathway for single-nucleotide polymorphism and restriction fragment presence/absence loci of hybrid catfish (*Clarias gariepinus*, Burchell, 1822 x *Clarias macrocephalus*, Günther, 1864) from a BLAST search of the genomes of Japanese rice fish (*Oryzias latipes*, Temminck and Schlegel, 1846), zebrafish (*Danio rerio*, Hamilton, 1822), Japanese puffer (*Takifugu rubripes*, Temminck and Schlegel, 1850), channel catfish (*Ictalurus punctatus*, Rafinesque, 1818) and chicken (*Gallus gallus*, Linnaeus, 1758) (70:30, male:female; XX/XY sex-determination type).

| Locus id | Gene^1,2^ | E-values | Query coverage | Similarity | Product | Function | Component | Pathway | Reference |
| --- | --- | --- | --- | --- | --- | --- | --- | --- | --- |
| PA55522914 | *SLC14A1* | 2 x 10^-15^ | 100% | 88.41% | Urea transporter 1 | Urea channel activity,  water transmembrane transporter activity | Cell membrane | Transmembrane transport | Levin et al. (2009) |
| PA55498173 | *FCRL5* | 0.005 | 94% | 75.38% | Fc receptor-like protein 5 | Transmembrane signalling receptor activity | Cell membrane | Cell surface receptor signalling pathway | Nakayama et al. (2001) |
| PA55522914 | *GRB2* | 4 x 10^-8^ | 69% | 88.89% | Growth factor receptor-bound protein 2 | Adapter protein that provides a critical link between cell surface growth factor receptors and the Ras signalling pathway | Cytoplasm, Nucleus, Endosome,  Golgi apparatus | epidermal growth factor receptor signalling pathway, fibroblast growth factor receptor signalling pathway | Lowenstein et al. (1992) |
| PA100005297 | *MEST* | 4 x 10^-15^ | 100% | 85.51% | Mesoderm-specific transcript homolog protein | Hydrolase activity | Endoplasmic reticulum membrane | Mesoderm development,  regulation of lipid storage | Gaudet et al. (2011) |
| PA35626399 | *FOXO3* | 0.001 | 71% | 82.00% | Forkhead box protein O3 | DNA binding, sequence-specific DNA binding, transcription regulatory region sequence-specific DNA binding | Nucleus, cytosol, Mitochondrion matrix | apoptotic process, cytokine-mediated signalling pathway, tumour necrosis factor-mediated signalling pathway | Peserico et al. (2013) |
| PA55532208 | *SIN3B* | 3 x 10^-4^ | 62% | 83.72% | SIN3 transcription regulator family member B | Chromatin binding,  transcription corepressor activity | Nucleus | Histone deacetylation, negative regulation of transcription by RNA polymerase II | Gaudet et al. (2011) |
|  |  |  |  |  |  |  |  |  |  |

^1^All genes show sequence similarity with a partial sequence of DArT loci of more than 60% from the NCBI database.

^2^All genes show an E-value with a partial sequence of DArT loci lower than 0.005 from the NCBI database.

Burchell, W. J. (1822). *Travels in the Interior of Southern Africa*. London: Creative Media Partners LLC.

Gaudet, P., Livstone, M. S., Lewis, S. E., and Thomas, P. D. (2011). Phylogenetic-based propagation of functional annotations within the gene ontology consortium. *Brief. Bioinform*. 12, 449–462. [doi: 10.1093/bib/bbr042](https://doi.org/10.1093/bib/bbr042)

Günther, A. (1864). *Catalogue of Fishes in the British Museum London.* London: Natural History Museum Press.

Levin, E. J., Quick, M., and Zhou, M. (2009). Crystal structure of a bacterial homologue of the kidney urea transporter. *Nature* 462, 757–761. doi: 10.1038/nature08558

Lowenstein, E. J., Daly, R. J., Batzer, A. G., Li, W., Margolis, B., Lammers, R., et al. (1992). The SH2 and SH3 domain-containing protein GRB2 links receptor tyrosine kinases to ras signaling. *Cell* 70, 431–442. doi: 10.1016/0092-8674(92)90167-b

Nakayama, Y., Weissman, S. M., and Bothwell, A. L. (2001). BXMAS1 identifies a cluster of homologous genes differentially expressed in B cells. *Biochem. Biophys. Res. Commun.* 285, 830–837. doi: 10.1006/bbrc.2001.5231

Peserico, A., Chiacchiera, F., Grossi, V., Matrone, A., Latorre, D., Simonatto, M., et al. (2013). A novel AMPK-dependent FoxO3A-SIRT3 intramitochondrial complex sensing glucose levels. *Cell. Mol. Life Sci.* 70, 2015–2029. doi: 10.1007/s00018-012-1244-6

**Supplementary Table S7** **|** Repeat searches for single-nucleotide polymorphic and restriction fragment presence/absence loci of hybrid catfish (*Clarias gariepinus*, Burchell, 1822 x *Clarias macrocephalus*, Günther, 1864) (XX/XY sex-determination type).

| Repeat^1^ | Type | PA loci | SNP loci | Total |
| --- | --- | --- | --- | --- |
| 70:30 male:female | | | | |
| DNA transposon | Tc1/mariner | 11 | - | 11 |
|  | hAT | 2 | - | 2 |
|  | Harbinger | 1 | - | 1 |
|  | MuDR | - | 1 | 1 |
| LTR retrotransposon | Gypsy | 5 | 1 | 6 |
| Non-LTR retrotransposon | L2 | 1 | - | 1 |
|  | *Rex1* | 1 | - | 1 |
| 80:20 male:female | | | | |
| DNA transposon | Tc1/mariner | 2 | - | 2 |

^1^LTR, long terminal repeats.

Burchell, W. J. (1822). *Travels in the Interior of Southern Africa*. London: Creative Media Partners LLC.

Günther, A. (1864). *Catalogue of Fishes in the British Museum London.* London: Natural History Museum Press.

**Supplementary Table S8 |** **S**ingle-nucleotide polymorphic and restriction fragment presence/absence loci of hybrid catfish (*Clarias gariepinus*, Burchell, 1822 x *Clarias macrocephalus*, Günther, 1864) inherited from bighead catfish (*Clarias macrocephalus*) and African catfish (*Clarias gariepinus*).

| Locus id | *Clarias macrocephalus* | *Clarias gariepinus* | Hybrid | Gene | TEs |
| --- | --- | --- | --- | --- | --- |
| HCAPA35630234 | 27:40 (male:female) | - | 87:20 (male:female) | - | DNA/Mariner |
| HCAPA55492443 | - | 93:47 (female:male) | 87:20 (male:female) | - | - |
| HCAPA35627549 | 93:87 (male:female) | - | 73:20 (male:female) | - | - |
| HCAPA35624731 | 93:100(male:female) | - | 73:27 (male:female) | - | - |
| HCAPA35630019 | 13:13 (male:female) | - | 73:27 (male:female) | - | - |
| HCAPA35626399 | 80:60 (male:female) | - | 73:27 (male:female) | - | - |
| HCAPA35629714 | 67:40 (male:female) | - | 73:27 (male:female) | - | - |
| HCAPA35641359 | 73:93 (male:female) | - | 73:27 (male:female) | - | - |
| HCAPA35627301 | 40:13 (male:female) | - | 80:13 (female:male) | - | - |
| HCAPA35636078 | 73:93 (male:female) | - | 73:13 (female:male) | - | - |
| HCAPA35640559 | 60:73 (male:female) | - | 73:13 (female:male) | - | - |
| HCAPA35627651 | 87:93 (male:female) | - | 87:20 (female:male) | - | - |
| HCAPA35643024 | 20:13 (male:female) | - | 80:20 (female:male) | - | - |
| HCAPA55516444 | - | 87:80 (female:male) | 80:20 (female:male) | - | - |
| HCAPA35626878 | 80:80 (male:female) | - | 73:20 (female:male) | - | - |
| HCAPA35641360 | 47:33 (male:female) | - | 73:20 (female:male) | - | - |
| HCAPA35636016 | 73:67 (male:female) | - | 73:20 (female:male) | - | - |
| HCAPA35624597 | 93:80 (male:female) | - | 73:20 (female:male) | - | DNA/Mariner |
| HCAPA55469998 | - | 87:80 (male:female) | 80:27 (female:male) | - | - |
| HCAPA35639998 | 47:53 (male:female) | - | 80:27 (female:male) | - | - |
| HCAPA35642856 | 67:47 (male:female) | - | 73:27 (female:male) | - | - |
| HCAPA35624997 | 47:67 (male:female) | - | 73:27 (female:male) | - | DNA/hAT |
| HCAPA35639366 | 27:20 (male:female) | - | 73:27 (female:male) | - | - |
| HCAPA35637844 | 13:33 (male:female) | - | 73:27 (female:male) | *B4GALNT1* | - |
| HCAPA35644970 | 47:20 (male:female) | - | 73:27 (female:male) | - | - |
| HCASNP35642896 | 13:13 (male:female) | - | 73:27 (female:male) | - | - |
| CMAPA35633505 | 73:07 (male:female) | - | 40:20 (female:male) | - | - |
| CMAPA35643628 | 73:07 (male:female) | - | 40:33 (female:male) | - | - |
| CMAPA35644321 | 73:07 (male:female) | - | 47:33 (female:male) | - | - |
| CMAPA35624424 | 100:13 (male:female) | - | 13:07 (male:female) | - | - |
| CMAPA35644497 | 80:13 (male:female) | - | 33:13 (male:female) | - | - |
| CMAPA35626310 | 73:13 (male:female) | - | 07:00 (female:male) | - | - |
| CMAPA35627304 | 73:13 (male:female) | - | 07:00 (male:female) | - | - |
| CMAPA35633283 | 73:13 (male:female) | - | 33:07 (male:female) | - | - |
| CMAPA35625943 | 73:13 (male:female) | - | 27:00 (female:male) | - | - |
| CMAPA35637761 | 100:20 (male:female) | - | 27:20 (female:male) | - | - |
| CMAPA35627190 | 93:20 (male:female) | - | 33:13 (female:male) | - | - |
| CMAPA35637373 | 87:20 (male:female) | - | 80:30 (female:male) | - | - |
| CMAPA35640034 | 87:20 (male:female) | - | 93:73 (female:male) | - | - |
| CMAPA35641059 | 87:20 (male:female) | - | 60:33 (female:male) |  | - |
| CMAPA35642640 | 87:20 (male:female) | - | 13:00 (male:female) | *ARFRP1* | - |
| CMAPA35644213 | 87:20 (male:female) | - | 53:13 (female:male) | - | - |
| CMAPA35629140 | 80:20 (male:female) | - | 53:20 (female:male) | - | - |
| CMAPA35633300 | 80:20 (male:female) | - | 27:07 (female:male) | *MED8* | - |
| CMAPA35644152 | 80:20 (male:female) | - | 33:33 (female:male) | - | - |
| CMAPA35624579 | 73:20 (male:female) | - | 13:07 (male:female) | - | - |
| CMAPA35626299 | 73:20 (male:female) | - | 53:27 (female:male) | - | - |
| CMAPA35642733 | 73:20 (male:female) | - | 20:07 (female:male) | - | - |
| CMAPA35644508 | 73:20 (male:female) | - | 33:20 (female:male) | *TFAP2B* | - |
| CMAPA35624378 | 73:20 (male:female) | - | 73:47 (female:male) | - | - |
| CMAPA35625718 | 73:20 (male:female) | - | 33:27 (female:male) | - | DNA/Mariner |
| CMAPA35635635 | 100:27 (male:female) | - | 67:60 (female:male) | *CHD3* | - |
| CMAPA35624487 | 93:27 (male:female) | - | 27:07 (female:male) | - | - |
| CMAPA35624538 | 87:27 (male:female) | - | 80:60 (female:male) | - | - |
| CMAPA35632713 | 87:27 (male:female) | - | 60:60 (female:male) | - | - |
| CMAPA35627245 | 80:27 (male:female) | - | 07:00 (male:female) | - | - |
| CMAPA35629269 | 80:27 (male:female) | - | 20:07 (male:female) | - | - |
| CMAPA35638320 | 80:27 (male:female) | - | 13:07 (female:male) | - | - |
| CMAPA35624614 | 80:27 (male:female) | - | 80:80 (female:male) | - | - |
| CMAPA35624823 | 80:27 (male:female) | - | 60:40 (female:male) | - | - |
| CMAPA35625208 | 80:27 (male:female) | - | 40:27 (male:female) | *CNOT4* | - |
| CMAPA35626949 | 80:27 (male:female) | - | 20:13 (female:male) | - | - |
| CMAPA35635054 | 80:27 (male:female) | - | 47:27 (male:female) | - | - |
| CMAPA35629319 | 80:27 (male:female) | - | 47:20 (female:male) | - | - |
| CMAPA35624375 | 73:27 (male:female) | - | 33:20 (male:female) | - | - |
| CMAPA35626069 | 73:27 (male:female) | - | 33:27 (male:female) | - | - |
| CMAPA35626619 | 73:27 (male:female) | - | 33:00 (male:female) | - | - |
| CMAPA35627441 | 73:27 (male:female) | - | 27:00 (female:male) | - | - |
| CMAPA35627590 | 73:27 (male:female) | - | 87:80 (female:male) | - | - |
| CMAPA35641871 | 73:27 (male:female) | - | 40:33 (female:male) | - | Gypsy |
| CMAPA35626005 | 73:27 (male:female) | - | 33:13 (male:female) | - | - |
| CMAPA35632238 | 73:27 (male:female) | - | 87:73 (female:male) | - | - |
| CMAPA35643978 | 80:07 (female:male) | - | 27:20 (male:female) | - | - |
| CMAPA35632705 | 80:13 (female:male) | - | 27:13 (female:male) | - | - |
| CMAPA35628544 | 73:13 (female:male) | - | 33:27 (female:male) | - | - |
| CMAPA35629937 | 73:13 (female:male) | - | 20:13 (female:male) | - | - |
| CMAPA35628922 | 87:20 (female:male) | - | 07:00 (female:male) | - | - |
| CMAPA35645212 | 80:20 (female:male) | - | 33:33 (female:male) | - | - |
| CMAPA35633492 | 73:20 (female:male) | - | 13:07 (male:female) | - | - |
| CMAPA35638739 | 73:20 (female:male) | - | 27:27 (female:male) | - | - |
| CMAPA35638741 | 73:20 (female:male) | - | 27:27 (female:male) | - | - |
| CMAPA35624931 | 93:27 (female:male) | - | 13:07 (female:male) | - | - |
| CMAPA35639260 | 93:27 (female:male) | - | 60:53 (female:male) | - | - |
| CMAPA35643748 | 87:27 (female:male) | - | 33:13 (male:female) | - | - |
| CMAPA35628738 | 80:27 (female:male) | - | 20:00 (male:female) | - | - |
| CMAPA35628630 | 80:27 (female:male) | - | 27:20 (female:male) | - | - |
| CMAPA35639120 | 80:27 (female:male) | - | 13:00 (male:female) | - | DNA/Kolobok |
| CMAPA35628034 | 73:27 (female:male) | - | 27:13 (female:male) | - | - |
| CMAPA35628625 | 73:27 (female:male) | - | 40:13 (female:male) | - | - |
| CMAPA35629966 | 73:27 (female:male) | - | 13:07 (male:female) | - | - |
| CMAPA35639520 | 73:27 (female:male) | - | 53:47 (male:female) | - | - |
| CMAPA35624836 | 73:27 (female:male) | - | 33:13 (male:female) | - | - |
| CMAPA35626639 | 73:27 (female:male) | - | 73:53 (female:male) | - | - |
| CMAPA35628302 | 73:27 (female:male) | - | 07:03 (female:male) | - | - |
| CMAPA35633136 | 73:27 (female:male) | - | 33:27 (female:male) | - | - |
| CMAPA35639148 | 73:27 (female:male) | - | 47:40 (male:female) | - | - |
| CMAPA35641063 | 73:27 (female:male) | - | 67:53 (male:female) | - | DNA/Mariner |
| CMASNP35643761 | 73:27 (male:female) | - | 20:13 (male:female) | - | - |
| CMASNP35635981 | 73:13 (female:male) | - | 27:00 (male:female) | *PHKA1* | - |
| CMASNP35643432 | 80:20 (female:male) | - | 07:00 (male:female) | - | - |
| CMASNP35633408 | 73:27 (female:male) | - | 07:00 (male:female) | - | - |

Burchell, W. J. (1822). *Travels in the Interior of Southern Africa*. London: Creative Media Partners LLC.

Günther, A. (1864). *Catalogue of Fishes in the British Museum London.* London: Natural History Museum Press.
